# A Single Protein S-acyl Transferase Acts through Diverse Substrates to Determine Cryptococcal Morphology, Stress Tolerance, and Pathogenic Outcome

**DOI:** 10.1371/journal.ppat.1004908

**Published:** 2015-05-13

**Authors:** Felipe H. Santiago-Tirado, Tao Peng, Meng Yang, Howard C. Hang, Tamara L. Doering

**Affiliations:** 1 Department of Molecular Microbiology, Washington University School of Medicine, St. Louis, Missouri, United States of America; 2 Laboratory of Chemical Biology and Microbial Pathogenesis, The Rockefeller University, New York, New York, United States of America; University of Rochester, UNITED STATES

## Abstract

*Cryptococcus neoformans* is an opportunistic yeast that kills over 625,000 people yearly through lethal meningitis. Host phagocytes serve as the first line of defense against this pathogen, but fungal engulfment and subsequent intracellular proliferation also correlate with poor patient outcome. Defining the interactions of this facultative intracellular pathogen with host phagocytes is key to understanding the latter’s opposing roles in infection and how they contribute to fungal latency, dissemination, and virulence. We used high-content imaging and a human monocytic cell line to screen 1,201 fungal mutants for strains with altered host interactions and identified multiple genes that influence fungal adherence and phagocytosis. One of these genes was *PFA4*, which encodes a protein S-acyl transferase (PAT), one of a family of DHHC domain-containing proteins that catalyzes lipid modification of proteins. Deletion of *PFA4* caused dramatic defects in cryptococcal morphology, stress tolerance, and virulence. Bioorthogonal palmitoylome-profiling identified Pfa4-specific protein substrates involved in cell wall synthesis, signal transduction, and membrane trafficking responsible for these phenotypic alterations. We demonstrate that a single PAT is responsible for the modification of a subset of proteins that are critical in cryptococcal pathogenesis. Since several of these palmitoylated substrates are conserved in other pathogenic fungi, protein palmitoylation represents a potential avenue for new antifungal therapeutics.

## Author Summary

*Cryptococcus neoformans* is a ubiquitous environmental yeast that kills over 625,000 people annually, mainly in developing countries. Healthy humans frequently inhale infectious particles without noticeable symptoms. However, in immunocompromised people, the initial lung infection can spread to other sites, particularly to the central nervous system where it causes lethal brain infection. The infected host responds by deploying immune cells to engulf and kill the yeast, but *C*. *neoformans* can survive this engulfment and even multiply within the host cells. To understand the interactions between the invading microbe and host cells we screened 1,201 fungal mutants to identify fungal factors that influence these processes. One mutant, lacking an enzyme that modifies proteins with the lipid palmitate, showed an increase in engulfment by the host along with dramatic defects in morphology, stress resistance, and virulence. We went on to identify the proteins this enzyme modifies and explain how its absence leads to altered cell wall synthesis, signal transduction, and membrane trafficking; these changes explain the behavior of the mutant. We also found that the mutant could not cause disease in an animal model. Our work shows that protein palmitoylation is critical for cryptococcal pathogenesis and presents a potential avenue for antifungal therapy.

## Introduction

*Cryptococcus neoformans* is a fungal pathogen that causes over 625,000 deaths per year, mainly in severely immunocompromised individuals. Cryptococcosis is contracted by inhalation of infectious particles from the environment [1], which leads to a primary pulmonary infection. In healthy people this infection is typically cleared, but in immunocompromised hosts the organism can proliferate and disseminate, with a tropism for the central nervous system where it causes lethal meningoencephalitis. As a result, this pathogen is a major threat to AIDS patients and to the rapidly growing population of individuals with other immunosuppressive conditions [2–5]. Host phagocytes, mainly macrophages, are critical for initial control of this facultative intracellular pathogen [6]. However, as the flip side to their positive role as the first line of host defense, these cells may also serve as sites for replication and latency, or potentially as vehicles for yeast dissemination [1]. In line with these activities, several studies have demonstrated a correlation between poor patient outcomes and the capacity of clinical strains to be phagocytosed and/or to proliferate intracellularly [7, 8]. Understanding the opposing roles of macrophages in cryptococcal infection and their interactions with *C*. *neoformans* is key to our ability to influence such events in favor of the host. Despite the importance of these interactions to cryptococcal pathogenesis, the critical features of the host and fungus that govern them have not been determined.

We developed an image-based high-throughput screening (HTS) assay to probe fungal-host cell interactions [9] and evaluated a *C*. *neoformans* partial deletion collection [10] for altered engulfment by a human macrophage-like cell line. One ‘hit’ lacked a gene that encodes a protein S-acyltransferase (PAT), incriminating protein palmitoylation as a key pathway in cryptococcal pathogenesis. Protein palmitoylation, the reversible addition of palmitate to cysteine, can regulate the stability, localization, and function of target proteins [11]. The enzymes mediating this modification were first identified in the model yeast *S*. *cerevisiae* [12, 13] and are now recognized as important effectors in eukaryotic cells [11]. Although protein palmitoylation has been shown to influence infectivity in viruses [14], bacteria [15], and parasites [16–18], its role in fungal pathogenesis has not been explored. The importance of this lipid modification in fungal pathogens is supported by studies of Ras1 localization in *Aspergillus fumigatus*, *C*. *neoformans*, and *Candida albicans* [19–21], but no other proteins have been shown to be functionally palmitoylated in these organisms. Finally, no PAT has been characterized in a pathogenic fungus. Our studies demonstrate that a single PAT is a major determinant of cryptococcal pathogenesis and, by defining the relevant palmitoylome, we identify the cellular mechanisms by which defects of this fatty acid modification dramatically alter fungal morphology, host interactions, and virulence *in vivo*.

## Results

### Identification of fungal genes that influence interactions with host macrophages

**Fig 1:**
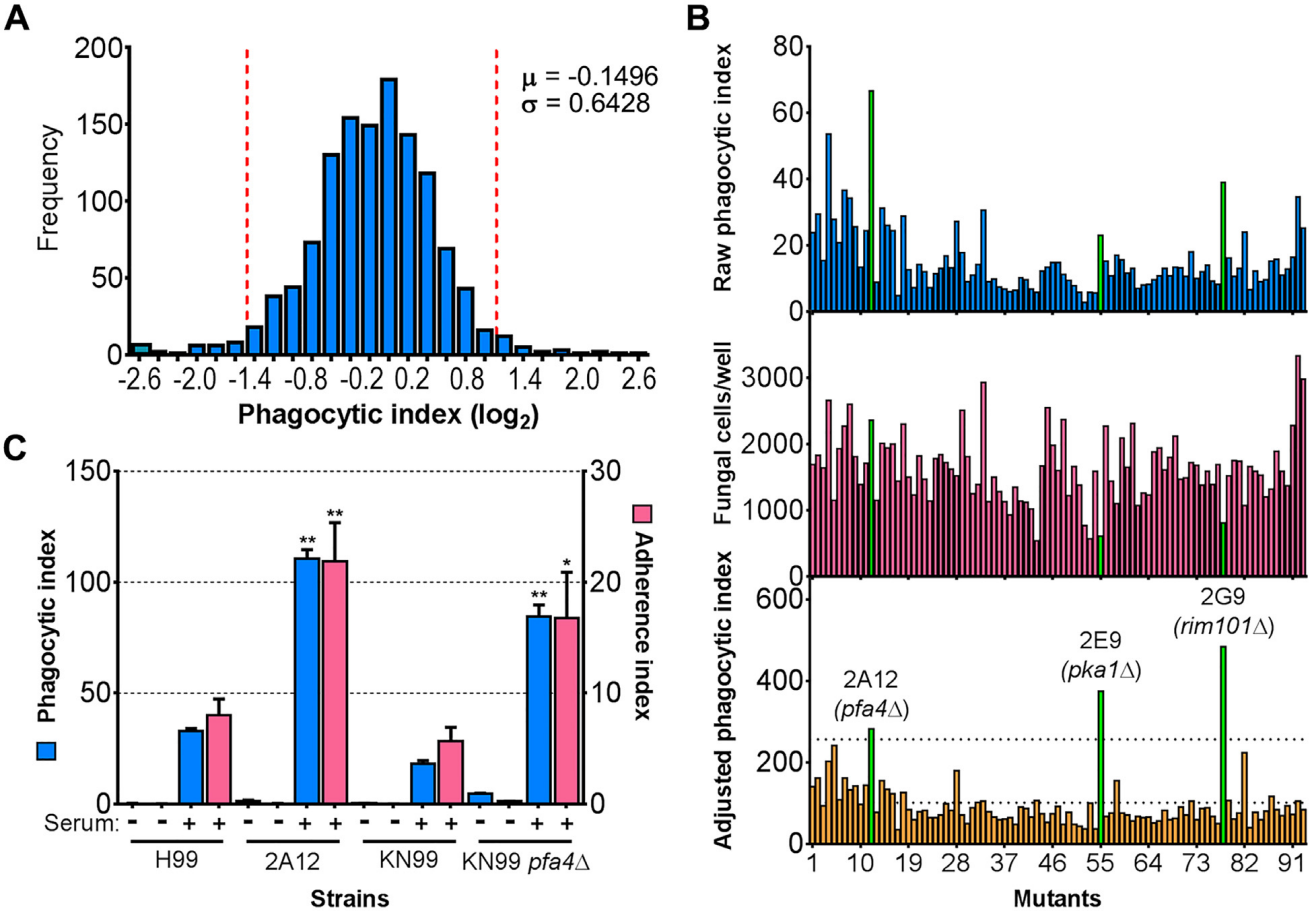
Identification of cryptococcal mutants with altered interactions with macrophages. (A) Distribution of 1,201 fungal mutants categorized by adjusted phagocytic index (fungi internalized/100 host cells, corrected for differences in inoculum; see Materials and Methods). Results were compiled from three independent replicate screens. Vertical dashed lines, two standard deviations (σ) above and below the mean (μ). (B) Plate 2 of the deletion collection (numbered 1 to 93) was assayed as in Materials and Methods. Shown are raw phagocytic index (top), *C*. *neoformans* counts in a parallel inoculum-only plate (middle), and adjusted phagocytic index (bottom). These results were representative of three independent replicate screens of this plate. Adjusted phagocytic indices of the three mutants indicated in green (*pfa4*Δ, *pka1*Δ, and *rim101*Δ) consistently exceeded our threshold (upper dotted line in the bottom graph) of two standard deviations above the plate mean (lower dotted line). This analysis shows only 93 of the Plate 2 strains: documentation for the mutant collection indicated that 2B2 was incorrect so it was omitted from the analysis and mutants 2B5 and 2E10 could not be recovered from the original plate. (C) Phagocytic and adherence indices for library strain 2A12 (*pfa4*Δ) and an independent *PFA4* deletion, each with its matched parental strain (H99 and KN99, respectively). All strains were screened ± serum opsonization as shown and mean values ± SEM are plotted. *, P < 0.05; **, P < 0.0001 compared to respective parent strain (Tukey’s multiple comparisons test).

*C*. *neoformans* engulfment by host cells and subsequent intracellular proliferation has been implicated in dissemination, virulence, and ultimately in patient outcome [8, 22, 23]. However, the full complement of fungal genes that participate in these processes has not been defined, and how individual gene products modulate interactions with host phagocytes is not known. To address cryptococcal interactions with host cells, we used an automated high content imaging method [9] to quantify the interactions between a human monocytic cell line (THP-1) and mutant fungi from a deletion collection made in the highly pathogenic reference strain H99 [10]. Of the 1,201 mutants we screened, 56 (4.7%) showed significant alterations in phagocytic index (Fig 1A). These mutants (S1 Table) were roughly equally distributed between strains with decreased and increased engulfment (30 and 26, respectively); the ten most extreme in each category are shown in Table 1. An example data set from one plate of the mutant collection (Fig 1B) shows strikingly increased phagocytosis of three mutants, two of which, *pka1* and *rim101*, are known to have altered cell surface structures that would explain this phenotype [24, 25]. Additionally, *pbx1*, the top hit of the high uptake mutants (Table 1), has defects in cell wall structure and capsule assembly that cause increased engulfment by macrophages [26]. These observations validated our strategy for probing the interactions between *C*. *neoformans* and macrophages and encouraged us to further pursue novel hits from our screen.

**Table 1:** *C. neoformans* mutants showing altered interactions with macrophages.^a^

Index (log_2_)^b^		Library well^c^	Gene ID	Gene name^d^	Biological role
**High Phagocytosis**	2.5	4C6	CNAG_01172	*PBX1*	Surface glycan synthesis and remodeling
	2.4	8E6	CNAG_02797	*CPL1*	Capsule synthesis and/or assembly
	2.3	2G9	CNAG_05431	*RIM101*	Transcription factor; regulation of cell wall assembly in response to pH
	2.1	11A5	CNAG_04514	*MPK1*	MAP kinase; cell integrity signaling and metabolite resistance
	2.1	9H11	CNAG_03018	***ASG101***	Zinc finger transcription factor; homologous to *S*. *cerevisiae ASG1*
	1.9	2E9	CNAG_00396	*PKA1*	cAMP dependent protein kinase; mating and virulence signaling
	1.8	12B2	CNAG_01551	*GAT201*	Transcription factor; regulation of anti-phagocytic mechanisms
	1.7	1A9	CNAG_06086	*CDK8*	Cyclin-dependent protein kinase 8
	1.5	10D3	CNAG_04863	*VPS25*	Component of the ESCRT complex; protein sorting/degradation
	1.4	8F11	CNAG_03188	*SET202*	Histone-lysine N-methyltransferase
**Low Phagocytosis**	-4.0	4H8	CNAG_01964	*OPT1*	Proton-coupled oligopeptide transporter
	-2.7	4C12	CNAG_01640	***CSF1***	Hypothetical protein; homologous to *S*. *cerevisiae CSF1*
	-2.6	9B5	CNAG_06759	***LPI1***	Dehydrogenase, similar to Zinc-binding oxidoreductases
	-2.5	5G8	CNAG_07351	***LPI2***	Hypothetical protein; no homologs in *S*. *cerevisiae*
	-2.4	4H9	CNAG_06370	*BAT2*	Branched-chain-amino-acid aminotransferase
	-2.2	12D6	CNAG_02580	***LPI3***	Hypothetical protein; no homologs in *S*. *cerevisiae*
	-2.0	9E4	CNAG_01262	*GPB1*	G-protein β-subunit involved in pheromone sensing and mating
	-2.0	9A12	CNAG_06074	***LPI4***	Cytoplasmic protein of unknown function
	-2.0	10H11	CNAG_00414	***MAK32***	Hypothetical protein; homologous to *S*. *cerevisiae MAK32*
	-2.0	1F6	CNAG_07534	***TRS130***	Hypothetical protein; homologous to *S*. *cerevisiae TRS130*

^a^ Top ten mutants with highest and lowest phagocytic index. See Supplementary S1 Table for a complete list.

^b^ Value shown is the average (on a binary log scale) of the adjusted uptake of each strain from three independent screens.

^c^ Location of the strain in the deletion collection (see Liu et al., 2008).

^d^ Bold indicates new names given to uncharacterized genes either based on homology to *S*. *cerevisiae* per nomenclature guidelines (see Inglis et al., 2014) or, for genes with no homology to *S*. *cerevisiae*, based on phenotype: *LPI* mutants, for Low Phagocytic Index. See Supplementary S1 Table for complete list and *HPI* mutants (High Phagocytic Index).

### A putative S-acyltransferase regulates *C*. *neoformans* uptake and adherence

Another strain (2A12) that consistently demonstrated an elevated phagocytic index (Fig 1B and Table 2) lacks the uncharacterized gene CNAG_03981. This gene is highly homologous to *S*. *cerevisiae PFA4*, which encodes a palmitoyl acyltransferase (PAT), and was accordingly given the same name (following guidelines in [27]). PATs are DHHC zinc finger domain-containing enzymes that mediate the reversible addition of palmitate to proteins, thereby regulating their membrane association and biological function [11]. Eukaryotic cells often express multiple DHHC domain proteins, which have similar enzymatic activity but modify variably overlapping groups of substrates [28]. These enzymes play key roles in protein fatty-acylation and membrane targeting [11], but have never been studied in *C*. *neoformans* or any other fungal pathogen. There are seven putative PATs encoded in the H99 genome; four of these were deleted in the collection that we screened but only *pfa4*Δ differed significantly from wild-type cells (Table 2). This suggested that Pfa4 acylates at least one protein that both influences host cell interactions and is not modified by other PATs. Given the limited knowledge of protein palmitoylation in *C*. *neoformans* biology and pathogenesis, we chose this mutant for mechanistic study.

**Table 2:** Comparison of putative PATs present on the deletion collection.

Index (log_2_)^a^		Library well^b^	Gene ID	Gene name	Description	*S*. *cerevisiae* homolog
**PATs**	1.142	2A12	CNAG_03981	***PFA4***	4 TMD palmitoyltransferase	*PFA4*
	0.005	6F1	CNAG_00274	Unnamed	4 TMD palmitoyltransferase	*SWF1/ERF2* ^c^
	-0.408	8A7	CNAG_00436	***AKR1***	6 TMD palmitoyltransferase with ankyrin repeats	*AKR1*
	-0.416	14H6	CNAG_02481	Unnamed	4 TMD palmitoyltransferase	*PFA3/PFA4* ^c^

^a^ Values are the average (on a binary log scale) of the adjusted uptake of each strain from three independent screens.

^b^ Location of the strain in the deletion collection (see Liu et al., 2008).

^c^ These *C*. *neoformans* genes do not have a single homolog in *S*. *cerevisiae*, they are most similar to the pair of genes indicated.

**Fig 2:**
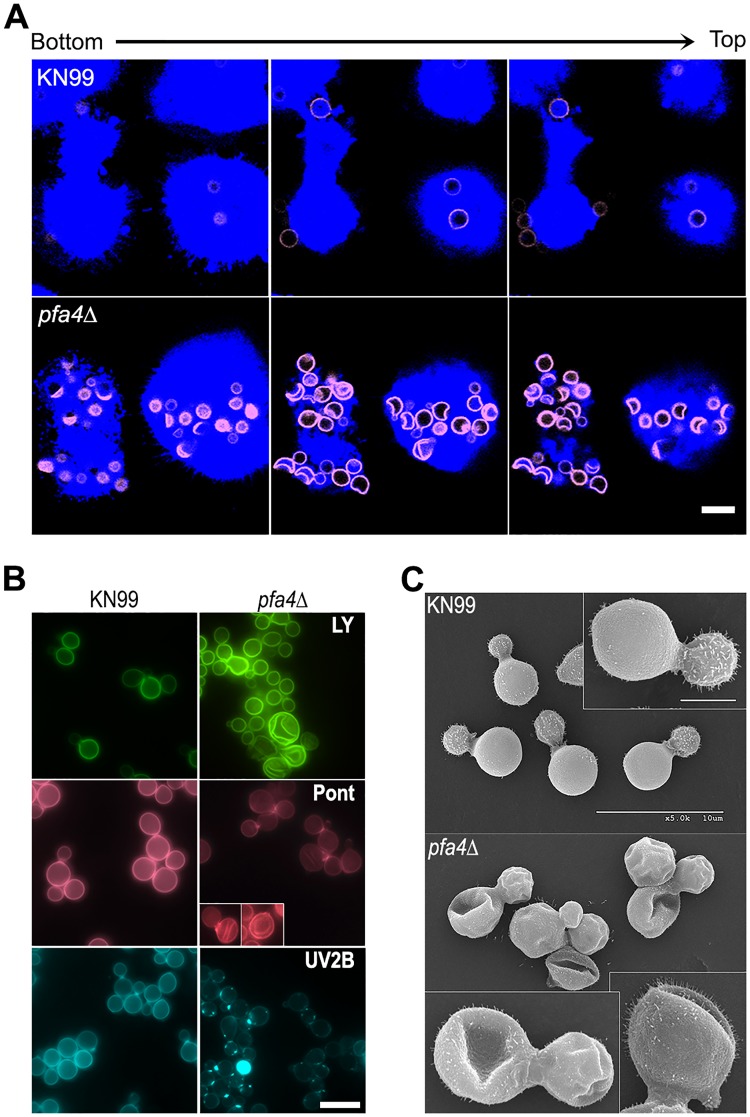
*pfa4*Δ mutant cells exhibit altered uptake by macrophages and morphological changes. (A) Confocal images of THP-1 cells exposed for 60 min to serum-opsonized wild-type (top) or mutant (bottom) fungi. Z-stack frames show bottom (side attached to the coverslip), middle, and top sections of the cells as also indicated by the horizontal arrow above the images. Corresponding movies are available as S1 and S2 Videos. Scale bar, 10 μm. (B) Representative fluorescent images of wild-type (left) and *pfa4*Δ (right) cells stained with Lucifer Yellow (LY), Pontamine (Pont), or Uvitex 2B (UV2B). Each pair of images was collected at the same settings, although brightness and contrast for the inset of Pont-stained *pfa4*Δ were enhanced to better show morphological defects. Scale bar, 10 μm. (C) SEM images of wild-type (top) and *pfa4*Δ (bottom) cells grown on YPD at 30°C. Similar images were obtained from two independent experiments using both H99 and KN99 genetic backgrounds. Scale bars on the upper panel (main figure, 10 μm; inset, 2.5 μm) also apply to the lower panel.

We first generated independent *pfa4* deletions in *C*. *neoformans* reference strain H99 (used for the deletion collection) and its more genetically tractable derivative KN99 [29]. Like 2A12, both mutants showed consistent increases in adherence to and engulfment by macrophages compared to wild-type cells (Fig 1C), with the greater uptake readily visible by confocal microscopy (Fig 2A and S1 and S2 Videos). These phenotypes, which were independent of the method used to label the cells (S1A and S1B Fig), were all reversed by complementation of the mutant with the wild-type gene at the endogenous locus.

The extremely high numbers of internalized mutant cells (Figs 1C and 2A) could potentially alter intracellular trafficking of *C*. *neoformans*, which is usually delivered to lysosomes after endocytosis [30]. To test this we used confocal microscopy to assess the progression of *pfa4*Δ and wild-type cells through various intracellular compartments after their exposure to host phagocytes (S2A Fig). The distribution of wild-type and mutant fungi between the cell surface (adherent cells), early endosomes (marked with EEA1), and lysosomes (marked with LAMP-1) was similar at late time points. The only significant differences were observed soon (15 min) after assay initiation, when a greater fraction of wild-type cells remained surface-accessible (adherent) while more mutant cells had already been phagocytosed (although not yet associated with EEA1). Overall, although the mutant is more efficiently internalized, both strains reach EAA1 and LAMP-1 compartments with similar dynamics. It has recently been suggested that *C*. *neoformans*-containing lysosomes do not completely acidify [31]. To test whether acidification differed between lysosomes containing *pfa4*Δ and wild-type yeast, we performed a phagocytosis assay in the presence of Lysotracker Red, a dye that becomes trapped and fluorescent in acidified organelles. We found that both strains were similarly distributed between unstained phagosomes and lysosomes (positive for Lysotracker; S2B and S2C Fig)

### *C*. *neoformans* lacking *PFA4* exhibits morphological defects and surface changes

In addition to an increased number of internalized *pfa4*Δ cells, our confocal studies revealed an unusual and dramatic change in their morphology (Fig 2A and S1 and S2 Videos). While wild-type cells are spherical, the mutant cells appeared to have collapsed in on themselves, manifested as membrane staining in either crescent shapes or double rings depending on cell orientation. This aberrant morphology occurred whether the fungi were inside macrophages (Fig 2A) or grown independently (Fig 2B), indicating that the alteration is intrinsic to the mutant rather than induced by the host cells. We tested other dyes to rule out the possibility that the shape change was due to the Lucifer Yellow (LY) stain used in our phagocytosis studies; in all cases we observed a similar phenotype (Fig 2B). Next, to eliminate the possibility that any compound that binds cell wall structures induces cell collapse, we imaged actively growing, unstained wild-type and *pfa4*Δ mutant cells by brightfield and differential interference contrast (DIC) light microscopy. Under these unstained, actively growing conditions we could easily detect the same aberrant shapes seen in *pfa4*Δ cells stained with various dyes (S3 Fig), indicating that they represent an intrinsic feature of this mutant. Finally, to get a detailed view of this morphological defect we examined the cells by scanning electron microscopy. Consistent with our light microscopy results, wild-type cells were globular and smooth while *pfa4*Δ cells were dramatically deformed (Fig 2C). Surprisingly, this has little effect on their ability to replicate at 30°C, where their growth rate is close to that of wild-type cells.

**Fig 3:**
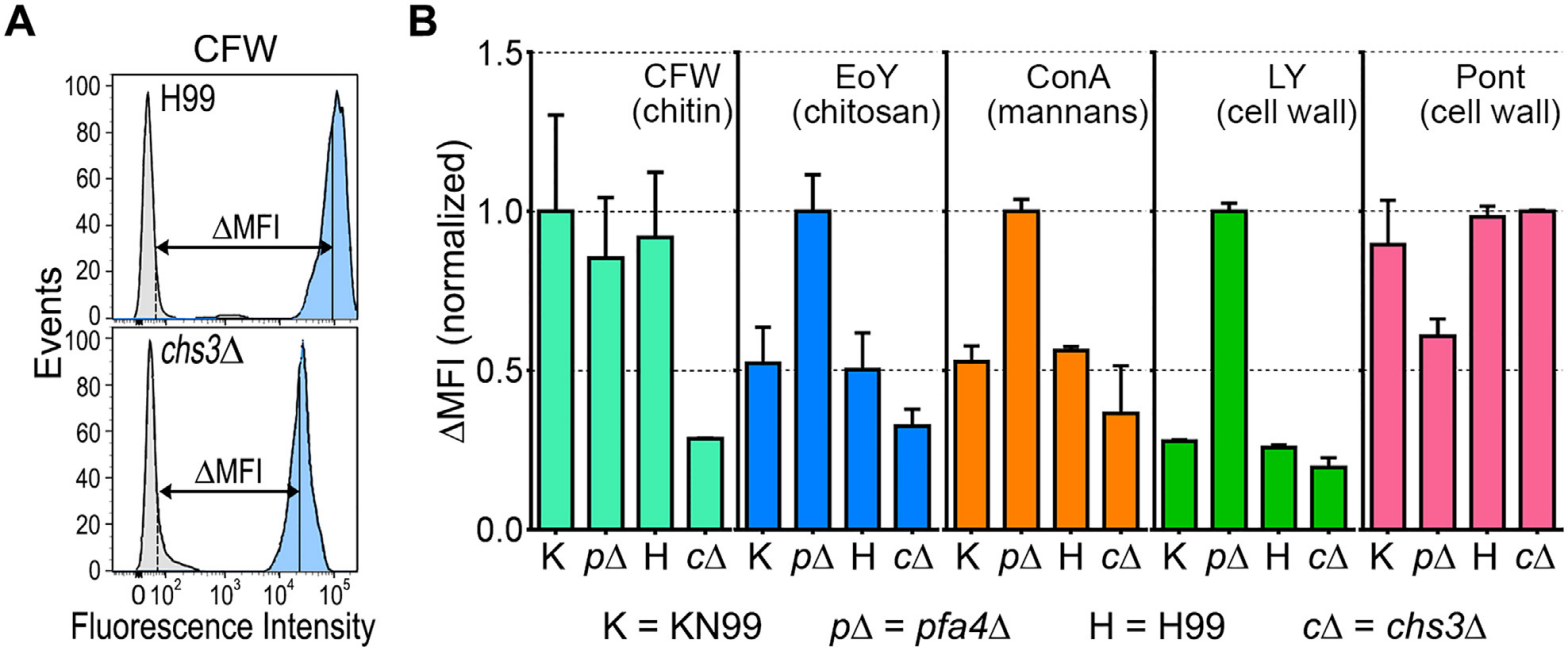
Exposure of cell surface components is altered in *pfa4*Δ cells. (A) Example of flow cytometry profiles used to assess the exposure/accessibility of cell wall components. Fluorescence intensity profiles of H99 and *chs3*Δ cells, either unstained (gray) or stained with calcofluor white (CFW; light blue) are overlaid to illustrate the difference in mean fluorescence intensity (ΔMFI). (B) ΔMFI for staining with CFW (binds chitin), Eosin Y (binds chitosan), Concanavalin A (binds mannoproteins), LY, and Pont (bind unspecified cell wall components); mean ± SEM of three independent experiments, with values normalized to the highest bar for each strain.

The *pfa4*Δ mutant showed altered initial interactions with host cells and aberrant morphology. One model that explains both observations is that the mutant has fundamental defects in cell wall structure that alter both surface molecule exposure and cell wall integrity. To probe cell wall organization, we used dye and lectin binding with flow cytometry to assess the accessibility of various cell wall components (Fig 3). We found that chitin accessibility, probed with calcofluor white (CFW), was not significantly altered in *pfa4*Δ, unlike the decreased signal in a chitin synthase mutant (*chs3*Δ) included as a control (Fig 3B). In contrast, probes of chitosan (Eosin Y; EoY) and mannans (Concanavalin A lectin; ConA) showed that these glycans were much more accessible in the *pfa4*Δ mutant (Fig 3B), supporting aberrant wall structure; this was also reflected in an altered staining pattern for ConA (S4 Fig). Similarly, LY and pontamine (Pont), also dyes that bind cell wall (although their specific targets are not defined), showed clear changes in binding the mutant compared to controls (Fig 3B). The abnormal exposure of chitosan and mannans at the surface of *pfa4*Δ cells could explain their greater recognition by macrophages (see Discussion).

**Fig 4:**
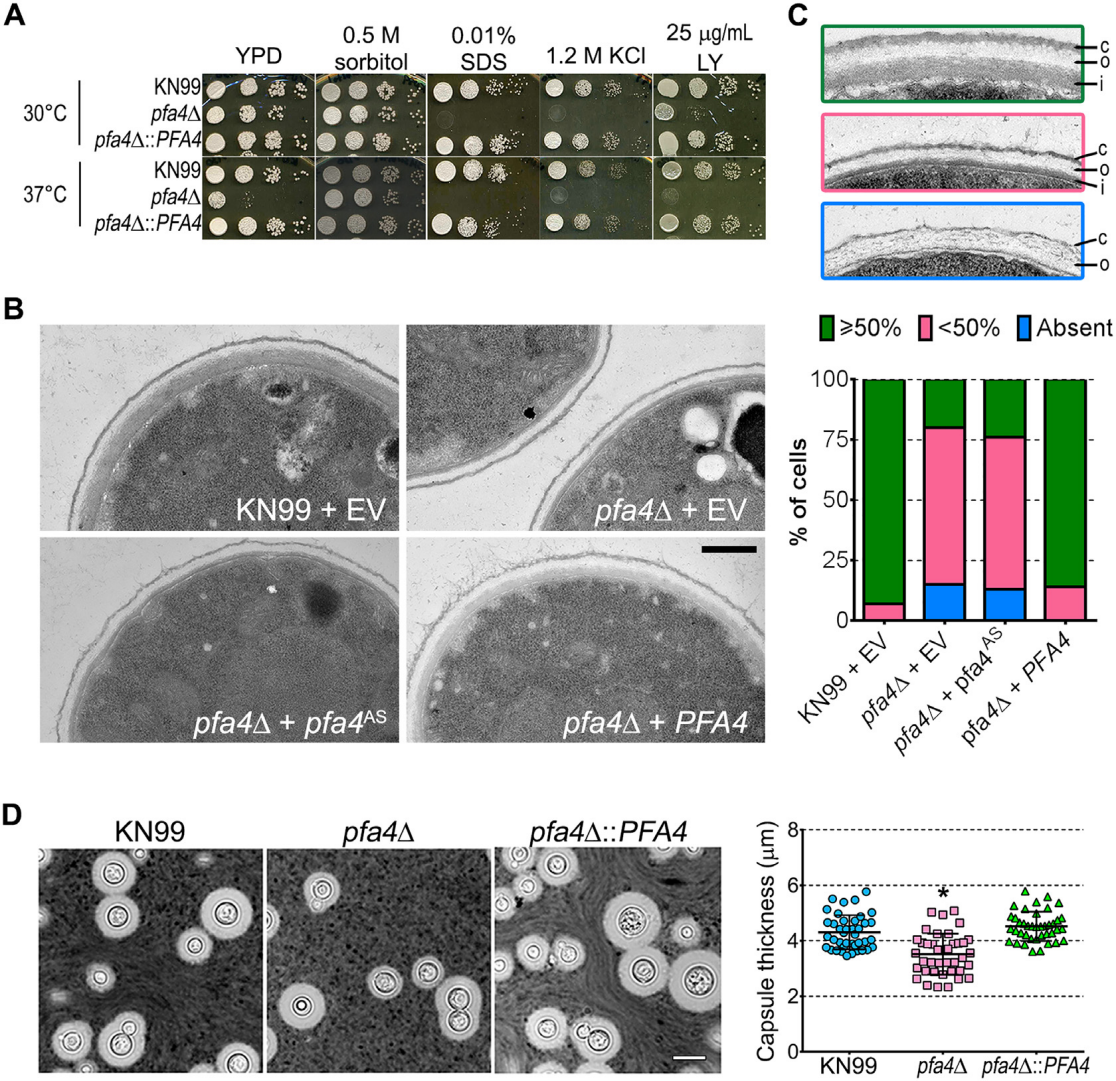
*pfa4*Δ has defects in cell wall integrity and structure. (A) 10-fold serial dilutions of the indicated strains on medium supplemented as shown. All plates were incubated for 3 days at either 30°C (top) or 37°C (bottom). (B) TEM of cells grown in YPD at 30°C. Each strain name is followed by the plasmid it carries: EV, empty vector; *pfa4* ^AS^, vector expressing catalytically-inactive Pfa4; *PFA4*, vector expressing wild-type Pfa4. Wild-type cells expressing mutant or wild-type *PFA4* looked like wild-type + EV. Scale bar, 500 nm. (C) Top, examples of normal and aberrant cell wall morphology; c, capsule; o, outer layer; i, inner layer. In normal cells (green outline) the inner layer was ≥50% of total wall thickness, while in mutants the inner layer was <50% (pink outline) or not visible (blue outline). Bottom, distribution of cell wall morphologies in various strains; only cells where the plasma membrane was clearly seen were measured. (D) Left, representative micrographs of the indicated strains, stained with India ink to show capsule. Right, capsule thickness of the same strains (individual data points and mean ± SD). *, P <0.0001 (Student’s t-test) comparing wild-type and mutant.

We reasoned that the altered arrangement of cell wall components in the *pfa4*Δ mutant would threaten overall cell integrity. We tested this hypothesis by plating serial dilutions of *pfa4*Δ in the presence of various stressors. Compared to wild-type and the complemented mutant, *pfa4*Δ was sensitive to plasma membrane damaging agents (SDS and H_2_O_2_), osmotic stress (KCl and NaCl), cell wall binding dyes (CFW, CR, and LY), and elevated temperature (37°C) (Figs 4A and S5). Only temperature sensitivity could be rescued by sorbitol (Fig 4A), suggesting that the cell integrity defects and temperature sensitivity are caused by perturbation of different pathways. This experiment also indicates that Pfa4 is not absolutely required for growth at high temperatures; in support of this conclusion, the *pfa4*Δ cells continued to grow slowly at 37°C for over a day even in the absence of sorbitol (S5A Fig). The mutant was also hypersensitive to treatment with cell wall lysing enzymes (S5C Fig), an assay which probes cell wall stability as well as cellular response to cell wall damage [32]. In all cases genomic or plasmid complementation of *pfa4*Δ restored wild-type phenotypes.

The pleiotropic effects of *PFA4* deletion suggested the dysfunction of one or more protein substrates of palmitoylation, which are not lipidated and therefore mislocalized, misfolded and/or degraded. To test whether the enzymatic activity of Pfa4 was indeed responsible for these phenotypes, we mutated its catalytic DHHC sequence to DHAS (S5D Fig); mutation of this cysteine abolishes PAT activity in other systems [12, 13, 16]. When both forms of the protein were expressed in *pfa4*Δ, only the wild-type rescued the mutant’s sensitivity to cell wall stress (S5E Fig), showing that the observed defects are due to a lack of PAT enzymatic activity.

The inability of *pfa4*Δ to withstand cell wall stress could reflect defects in cell wall structure, inability to respond to and repair a damaged wall, or both. To investigate cell wall structure we used transmission electron microscopy (TEM). The walls of wild-type strains and of *pfa4*Δ expressing wild-type *PFA4* were fairly uniform in thickness, and showed the expected multilayered organization [33]: an electron-dense inner layer surrounded by a more electron-lucent layer and then an outer rim of capsule (Fig 4B; the capsule layer is thin because the strains were grown in rich medium). In these cells the inner layer was always ≥50% of the total wall width (example shown in Fig 4C, top image). In contrast, the cell walls of the mutant (with or without the catalytically-dead Pfa4^AS^) were generally thinner, primarily due to a reduction in the inner layer (Fig 4B and 4C, middle image). In ~80% of these cells the inner layer was <50% of the total wall width or was completely absent (Fig 4C, graph); in many of them the existing outer layer was also disorganized (Fig 4C, bottom image).

We next tested whether *pfa4*Δ cells have defects in cell wall stress signaling that render them unable to respond to environmental changes, by growing serial dilutions of wild-type, mutant, and mutants expressing either *PFA4* or the catalytically-dead *pfa4* ^AS^ on media containing caffeine (S5E Fig). Caffeine stimulates the cAMP/PKA pathway, activating *PKA1/2* and thereby mimicking cell wall stress. This chemical activation of the cell integrity pathway can be lethal if there is a preexisting defect in the pathway [34]. *pfa4*Δ could not grow under these conditions, consistent with a signaling defect in response to cell wall stress. Taken together, these results indicate that *pfa4*Δ cells have both altered cell wall structure and defective transduction of signals from the cell integrity pathway that would normally compensate for such changes. This results in a disordered wall with altered exposure of cell wall components, which in turn likely facilitates recognition by host cells (see Discussion).

A distinguishing feature and major virulence factor of *C*. *neoformans* is its polysaccharide capsule, which associates with the cell wall via α-glucan [33, 35]. We observed that *pfa4*Δ cells were clumpy in culture, a characteristic often seen in hypocapsular cryptococci that suggested these cells might have a capsule defect. Interestingly, this was not observed: the capsules of *pfa4*Δ cells were morphologically similar to those of wild-type under inducing conditions, although they were slightly smaller overall (Fig 4D).

### Pfa4 is required for *in vitro* and *in vivo* virulence

**Fig 5:**
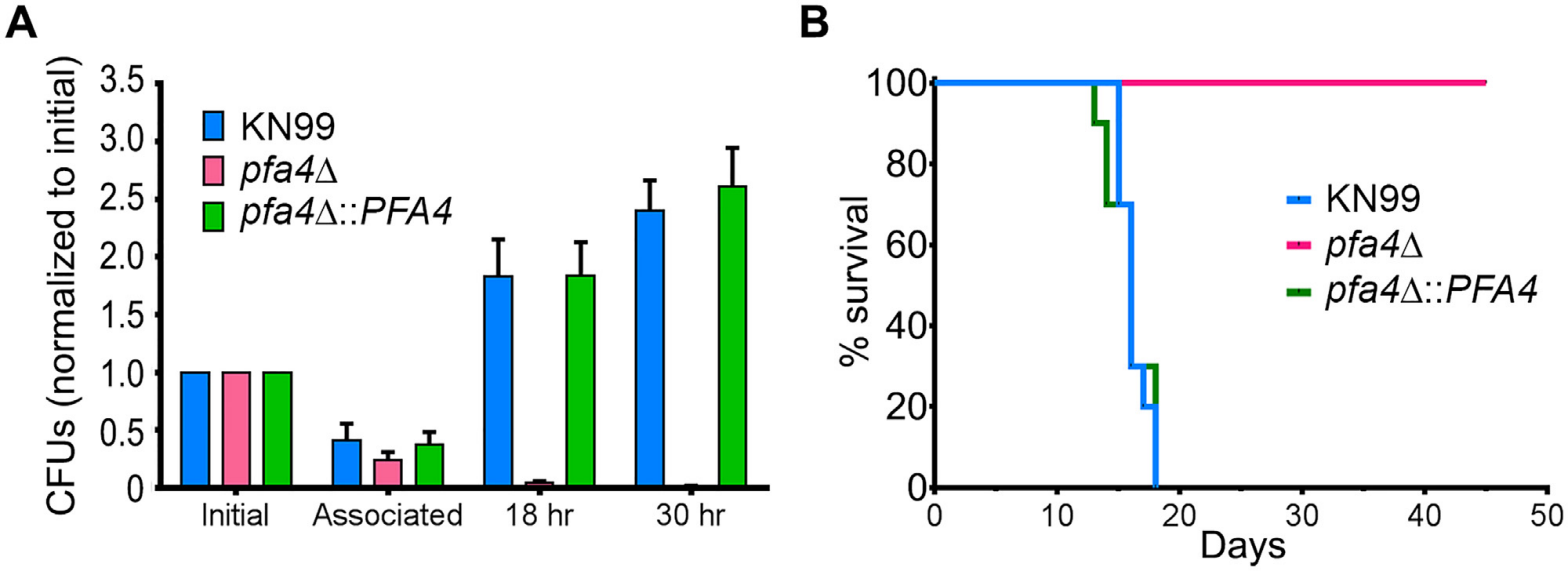
*pfa4*Δ is avirulent *in vitro* and *in vivo*. (A) Fungi and THP-1 cells were co-incubated for 1 hr at MOI ≤ 1 and then washed vigorously to remove free cryptococci. THP-1 cells were lysed for assessment of CFU immediately after washing (denoted as ‘associated’) and at two subsequent time points. Averages + SEM compiled from three independent experiments are plotted relative to the initial inoculum. (B) 10 AJ/Cr mice per group were infected intranasally with 5 x 10^4^ *C*. *neoformans* and monitored for up to 45 days. The inocula used for nasal inhalation for each group were verified by spotting in YPD agar.

*C*. *neoformans* survives and proliferates within macrophage phagolysosomes [31, 36, 37]. We assessed the behavior of *pfa4*Δ cells in this challenging environment and found that host phagocytes rapidly killed them (Fig 5A). In contrast, wild-type and complemented mutant cells showed robust growth in this context (Fig 5A), and even caused host cell numbers to decrease slightly (they were unperturbed by the *pfa4*Δ mutant).

We further tested the virulence of *pfa4*Δ in a mouse model of cryptococcosis, monitoring disease progression by weight loss. Infection with wild-type or the complemented mutant killed 50% of the mice in 16 days, with all animals steadily losing weight by about two weeks and succumbing to infection by day 18 (Fig 5B). In contrast, mice infected with *pfa4*Δ showed a modest (3–5%) and transient (days 8–14) weight loss early in infection, but recovered and grew normally until the study was terminated at day 45; no CFU were recovered from lung or brain at that time. This dramatic effect of a single PAT on fungal pathogenesis is unprecedented.

### Identification of Pfa4-specific substrates

**Fig 6:**
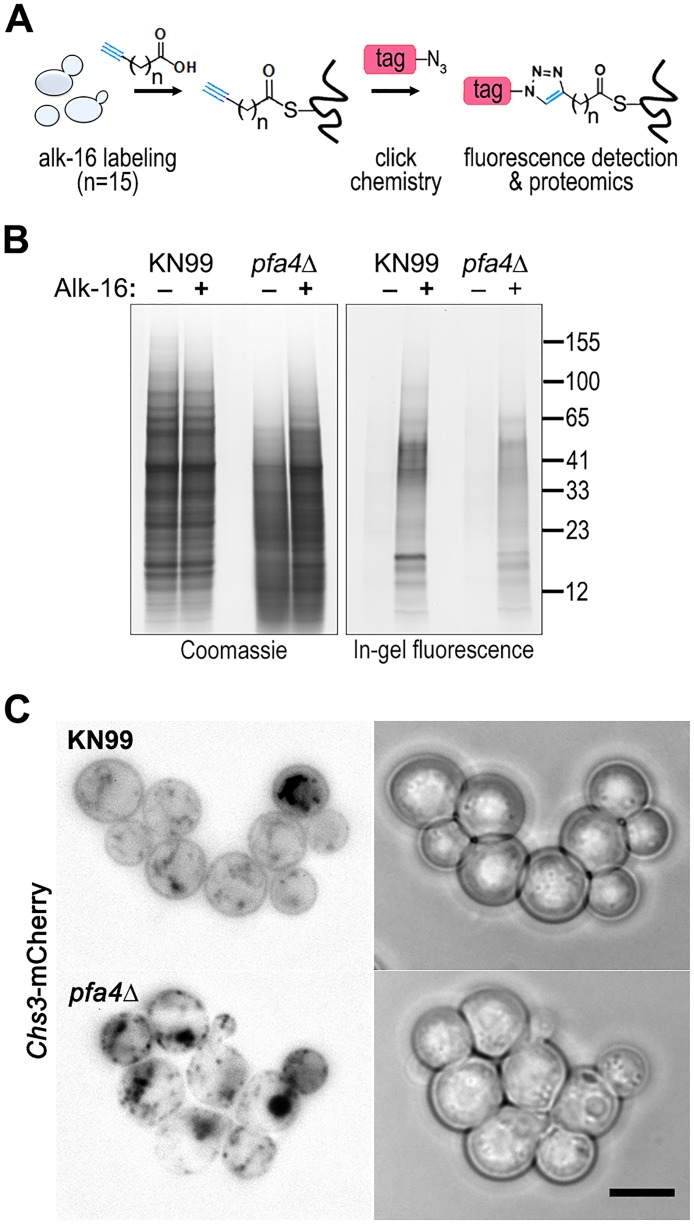
Identification of Pfa4-specific substrates and Pfa4-dependent Chs3 localization. (A) Schematic depiction of bio-orthogonal labeling of proteins with alk-16 and an azido-reporter (tag, azido-rhodamine for fluorescence detection or azido-biotin for affinity purification). (B) Total proteins from wild-type and mutant cells labeled ± alk-16, resolved by SDS-PAGE, and analyzed by Coomassie stain or in-gel fluorescence. MW standards (in kDa) are indicated on the right. (C) Localization of Chs3-mCherry expressed in the strain indicated and visualized by fluorescence (left, shown as a negative image for clarity) or brightfield microscopy (right). Scale bar, 5 μm.

Despite the importance of palmitoylation to fundamental processes of cell biology [11], the palmitoylome of *C*. *neoformans*, like that of other fungal pathogens, has never been defined, with only one protein (Ras1) shown to be functionally palmitoylated [19]. The dramatic effects of *PFA4* deletion, which our active-site mutation studies showed are due to lack of enzymatic activity, indicate that this lipid modification is crucial for cryptococcal cell integrity and virulence. To mechanistically explain these observations, we used fatty acid chemical reporter labeling and bioorthogonal chemistry proteomics to determine the specific set of proteins modified by Pfa4 [38–40]. In this method, cells are metabolically labeled with alk-16, a palmitic acid analog with an alkyne group, which is incorporated into proteins in place of the normal fatty acid (Fig 6A). Proteins modified with alk-16 can then be labeled with azide-functionalized reagents via ‘click chemistry’ for fluorescence detection or proteomic analysis (Fig 6A; [39–41]). We grew wild-type and *pfa4*Δ cells with alk-16, and then performed labeling reactions with azido-rhodamine for in-gel fluorescence detection (Fig 6B; [38]). The total protein profile of the mutant was similar to that of wild-type, although there were fewer species at high molecular weights; alk-16 labeling did not alter this pattern. The alk-16-labeled proteins of both strains also showed similar profiles, but with a slight decrease in the overall levels of modified proteins in the mutant (Fig 6B).

We next reacted alk-16-labeled proteins with azido-biotin, purified the biotinylated proteins with streptavidin beads, and evaluated Pfa4-specific alk-16-labeled proteins by comparative proteomics. Of the 427 proteins identified in two independent experiments with at least 2 unique peptides, 72 showed ≥5-fold enrichment in the wild-type compared to the *pfa4*Δ mutant in both studies (S1 File). High-confidence Pfa4 substrates included proteins that act in a variety of cell wall processes, including cell wall synthesis, membrane trafficking, signal transduction, and transport (Table 3). At the top of our list was chitin synthase 3 (Chs3), which has been characterized as a Pfa4 substrate in *S*. *cerevisiae* [42, 43]. Interestingly, a second chitin synthase (Chs1) is also a Pfa4 substrate.

**Table 3:** *C. neoformans* palmitoylated proteins enriched in wild-type over *pfa4*Δ samples.^a^

**Accession number**	Gene ID	Gene name	Description^b^	*S*. *cerevisiae* gene (Y/N)^c^
**Cell wall synthesis**				
J9VXM5	CNAG_05581	*CHS3*	Major chitin synthase (class IV)	*CHS3* (Y)
O13356	CNAG_03099	*CHS1*	Minor chitin synthase (class IV)	*CHS3* (Y)
**Membrane trafficking**				
J9W480	CNAG_05615	Unnamed	Plasma membrane t-SNARE (Syntaxin 1B)	*SSO1/2* (Y)
J9VSG0	CNAG_04484	Unnamed	Hypothetical protein; contains the Uso1/P115-like domain, present in vesicle tethering proteins	*USO1* (Y)
J9VP42	CNAG_05933	Unnamed	Hypothetical protein; contains a Sec1-like domain, implicated in vesicle docking and exocytosis	*SLY1* (N)
**Signal transduction**				
T2BQF0	CNAG_00556	*CCK1*	Casein kinase I	*YCK1/2* (Y)
J9VX74	CNAG_04505	*GPA1*	Guanine nucleotide-binding protein (Large G-protein) subunit alpha	*GPA2* (Y)
J9VN71	CNAG_06606	*RHO11*	Rho family GTPase (one of three similar to ScRho1)	*RHO1* (N)
J9VPP9	CNAG_02458	Unnamed	GTPase activating protein (Rho-GAP)	*RGD2* (Y)
J9VGM9	CNAG_03796	Unnamed	Similar to NAK-protein kinases (serine/threonine kinases)	*ENV7* (Y)
**Membrane transporters**				
J9VUI4	CNAG_01683	*STL1*	Putative monosaccharide transporter	*STL1* (N)
J9VKM7	CNAG_03664	*NIC1*	Major nickel transporter	None
J9VHU4	CNAG_00815	*SIT1*	Siderophore iron transporter	*SIT1* (N)
J9VNQ3	CNAG_03824	Unnamed	Mitochondrial phosphate transporter	*MIR1* (Y)
**Other**				
J9VIT9	CNAG_00354	VAC8	Vacuolar protein 8	*VAC8* (Y)
J9VJV1	CNAG_02981	*SIN3a*	Paired amphipathic helix protein; contains domains found on transcriptional regulators	*SIN3* (Y)
J9VMS8	CNAG_00854	*ERG2*	C-8 sterol isomerase	*ERG2* (N)
J9VVE7	CNAG_02129	Unnamed	Hypothetical protein; contains domain of unknown function unique to fungi	None
J9VVG0	CNAG_02114	Unnamed	Hypothetical protein; contains fungal-specific SUR7 domain	None
J9VKY1	CNAG_01010	Unnamed	Hypothetical protein; similar to mitochondrial transporters	None
J9VWW5	CNAG_04383	Unnamed	Acetyltransferase	None

^a^ Top high confidence hits identified in the proteomics analysis as Pfa4-specific substrates.

^b^ If the protein function has not been reported, the description is based on annotations in FungiDB (www.fungidb.org) for the corresponding gene.

^c^ Gene name of closest *S*. *cerevisiae* homolog, along with whether the protein was found to be palmitoylated (Y) or not (N) in a global analysis of palmitoylation in *S*. *cerevisiae* (Roth et al., 2006).

A number of substrates from our Pfa4-dataset have homologs that are known to be palmitoylated in *S*. *cerevisiae*, although not necessarily by Pfa4; these include Sso1 and Sso2, Vac8, Gpa2, Yck1 and Yck2, and Env7 [28]. Some have homologs known to be palmitoylated in other systems, such as Rho11 [44] and Vac8 [16]. Notably, *C*. *neoformans* Ras1 was labeled by alk-16 independent of Pfa4 (S1 File). This is consistent with previous studies in budding and fission yeast demonstrating that Ras1 is also a substrate of the Erf2/4 PAT complex, which is intact in our mutant [28, 41, 45]. Together, our results indicate that Pfa4 does not significantly alter global levels of fatty-acylation in *C*. *neoformans*, but palmitoylates specific proteins central to stress resistance and consequently to virulence, despite the presence of six other probable PAT genes in the cryptococcal genome.

**Fig 7:**
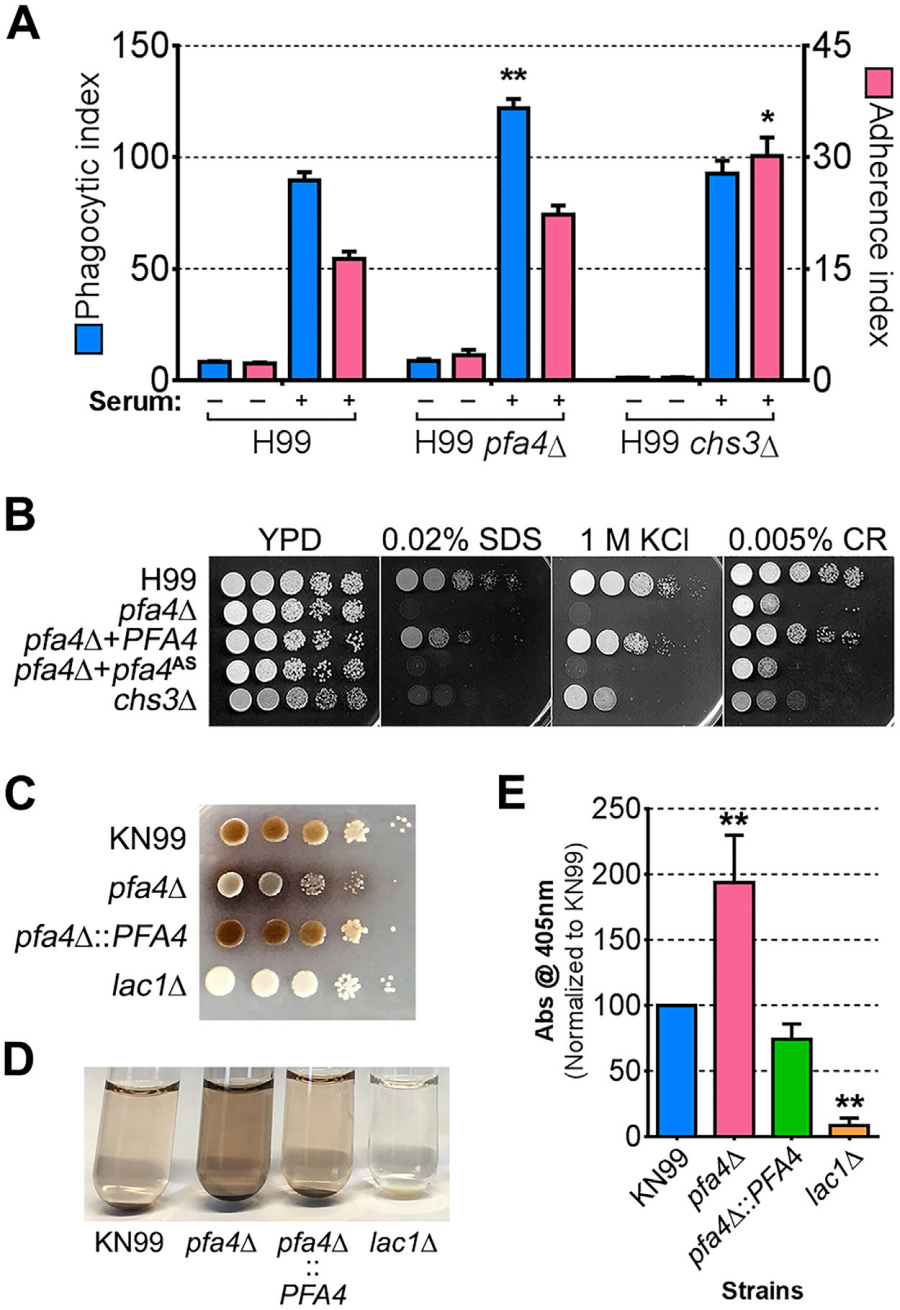
Phenotypic comparison of *pfa4*Δ and *chs3*Δ cells. (A) THP-1 uptake assay. Adherence and engulfment of wild-type, *pfa4*Δ, and *chs3*Δ strains were assayed as in Fig 1. *, P < 0.05; **, P < 0.0001 compared to H99 control (Tukey’s multiple comparisons test). (B) 10-fold serial dilutions of the indicated strains were grown at 30°C on the indicated media. (C) 10-fold serial dilutions of the indicated strains were spotted on L-DOPA medium for detection of melanin. Melanin released into the medium is visible as a dark halo. (D) Melanin release into liquid medium. Cultures of the strains indicated were grown for 18–24 hr in glucose-free asparagine medium containing L-DOPA (see Materials and Methods), subjected to centrifugation, and photographed. The image shown is representative of three independent experiments, each done in duplicate or triplicate. (E) Quantitation of released melanin in the supernatant fractions from (D). Shown are the averages ± SD of all three experiments. **, P < 0.0001 compared to KN99 control (Dunnett's multiple comparisons test). The *lac1*Δ strain was used as a negative control for melanin production in panels C-E.

Chs3 is critical for normal wall synthesis and maintenance [32, 46]. The discovery that it is a major substrate of Pfa4 is consistent with the multiple cell wall-related defects we observed in the *pfa4*Δ mutant, and explains how Pfa4 influences cell morphology, integrity, and consequently virulence. To establish a direct link between Pfa4-mediated palmitoylation and Chs3 function, we generated strains expressing Chs3-mCherry from the endogenous locus in both wild-type and *pfa4*Δ backgrounds. Palmitoylated Chs3 localized to internal compartments and to the plasma membrane, seen as a homogeneous rim outlining the cells (Fig 6C, top panel). In contrast, in the *pfa4*Δ cells, Chs3 is restricted to internal membranes, with occasional staining of vacuoles, suggestive of degradation (Fig 6C, bottom panel). This mislocalization of Chs3 is consistent with lack of palmitoylation of this protein and the cell wall-related defects observed in *pfa4*Δ cells. Interestingly, *pfa4*Δ and *chs3*Δ cells do not have completely congruent phenotypes. For example, *chs3*Δ does not exhibit the increased phagocytosis that first brought *pfa4*Δ to our attention (Fig 7A), although both strains show increased sensitivity to some cell wall stressors (Fig 7B) and poor retention of melanin at the cell wall (Fig 7C–7E, and [32]). Differences between the two mutants are likely due to the redundancy of both chitin synthases and palmitoyltransferases in *C*. *neoformans*, as well as the reduced palmitoylation of other Pfa4 substrates in the *pfa4*Δ mutant (see Discussion).

## Discussion

Phagocytes play multiple roles in cryptococcal pathogenesis, destroying fungi under some circumstances but also potentially harboring them and enabling them to survive, proliferate, and disseminate [1, 36]. Some outcomes of cryptococcal interactions with macrophages, including fungal engulfment and intracellular proliferation, correlate highly with patient outcome [7, 8]. These observations make host-pathogen interactions a compelling area of study, and raise the question of whether they might present feasible targets for antifungal therapy. Pursuing this question, however, requires mechanistic understanding of these events from the vantage point of both host and pathogen.

As a first step in such investigations, we used a high-content imaging-based assay to screen 1,201 *C*. *neoformans* mutants (corresponding to ~17% of the genome). We found 56 mutants that showed significantly altered uptake by host cells, including 29 lacking genes of unknown function that have not previously been investigated. Many of the mutants showing increased engulfment had been reported to be defective in host-pathogen interactions in other models; this validated our screen and provided strong support for uncharacterized hits. The genes deleted in several of the high-uptake mutants encode proteins involved in synthesis or remodeling of the cell wall and/or capsule, surface structures that interact most directly with host cells. Others encode signaling molecules or transcription factors involved in the response to environmental changes, such as would be encountered during infection. Intriguingly, most of the hits with reduced engulfment, more than half of which encode proteins with no known homologs in *S*. *cerevisiae*, have never been investigated. Future studies defining their biological roles should increase our understanding of *C*. *neoformans*’ interactions with host cells. Notably, the level of engulfment has no simple relationship to overall virulence in animal models, perhaps illustrating the complex role of phagocytosis in cryptococcal infection [36, 47]. For example, two hypervirulent mutants [10] showed opposite uptake results, with one (9A12) very poorly internalized while the other (2G9; lacking *RIM101*) was avidly engulfed.

One mutant that demonstrated increased uptake by phagocytes lacks *PFA4*, which encodes a protein containing the well-characterized DHHC domain characteristic of PAT enzymes. PATs catalyze the post-translational addition of palmitate to proteins, a reversible modification that can influence the localization, stability, and/or function of their substrates. The *C*. *neoformans* H99 genome contains seven genes encoding DHHC-domain proteins, and functional redundancy is common in this family of enzymes. It was therefore surprising that deletion of *PFA4* had such a dramatic effect on *C*. *neoformans* morphology, stress sensitivity, and virulence. This suggested that Pfa4 modifies specific substrates that are critical in cryptococcal biology. For this reason, and because of the recent attention to PATs as potential antimicrobial drug targets [48, 49], we investigated the mechanism(s) by which lack of Pfa4 causes these phenotypes.

We postulated that Pfa4 was the primary or sole PAT modifying important cryptococcal proteins required for cell integrity and virulence. Our proteomic analysis supported this hypothesis, identifying 72 proteins as preferentially palmitoylated by Pfa4 (Table 3 and S1 File). As in *S*. *cerevisiae* [42], Chs3 is a key Pfa4 target. This protein is one of eight cryptococcal chitin synthases and is responsible for synthesizing the majority of cellular chitin during vegetative growth [32, 46]. If Chs3 does not properly localize and act in *pfa4*Δ cells as a result of lacking palmitoylation, one would expect to see cell walls with reduced chitin and consequently impaired function. This is exactly what we observe: Chs3-mCherry in the mutant is mostly restricted to internal membranes and is depleted from the plasma membrane compared to in WT cells (Fig 6C); as a consequence, the inner layer of the cell wall, which corresponds to the layer containing chitin [33], is markedly reduced. Chs1, another class IV chitin synthase, is also preferentially modified by Pfa4 and may contribute to these cell wall defects.

Beyond altered chitin synthase activity, cell wall production is likely further compromised in *pfa4*Δ cells secondary to defects in intracellular traffic. Pfa4 substrates that we identified include several proteins involved in protein secretion that are known to be palmitoylated in *S*. *cerevisiae* (Table 3) or other organisms. Since multiple proteins involved in cell wall biogenesis are membrane proteins that travel to their site of action in secretory vesicles, dysfunction of SNARES or other proteins involved in this transport could alter cell surface composition via partial blockade or mislocalization of vesicle cargo.

Aberrant cell wall synthesis probably causes the dramatically altered morphology of *pfa4*Δ cells (Figs 2 and S3). Such changes were previously only seen in dying cryptococci that had been exposed to harsh conditions, such as digestion with lysosomal extracts *in vitro* or extended growth in infected animals [50, 51]. In contrast, *pfa4*Δ shows wall collapse even during normal growth in culture in the absence of any stains or exogenous compounds. Mutant cells are also hypersensitive to salt and sorbitol, suggesting defects in regulating turgor pressure. Regulatory disturbance is further suggested by the sensitivity of *pfa4*Δ to caffeine, which activates the cell integrity pathway. These phenotypes are consistent with our identification of several proteins that function in signal transduction as Pfa4 substrates (Table 3). These include Rho11, a GTPase that acts in cell integrity signaling via the MAP kinase pathway [52], and an uncharacterized protein similar to Rho GTPase activating protein (GAP) that may be the Rho11 GAP. Mislocalization of these proteins would likely impair the cellular response to cell wall damage. We also identified the α subunit of the large G-protein Gpa1 as a Pfa4 substrate; this protein is upstream of cryptococcal cAMP signaling and is involved in pheromone and mating responses [53], which could explain the mating defects when *pfa4*Δ strains are crossed to each other (S6 Fig). Perturbation of multiple signaling pathways due to lack of Pfa4 severely limits the mutant cells’ ability to respond appropriately to changing environmental conditions, exacerbating the effects of defective wall synthesis and undermining mutant survival in the host.

We considered the possibility that the increased uptake of *pfa*4Δ cells by host phagocytes reflected inviability of the yeast. However, we ruled out this possibility by demonstrating viability of the mutant under conditions of our uptake assays (S5 Fig). Furthermore, killing fungi by treatment with heat, ethanol, or azide did not alter uptake of wild-type (S1C Fig) or mutant cells.

**Fig 8:**
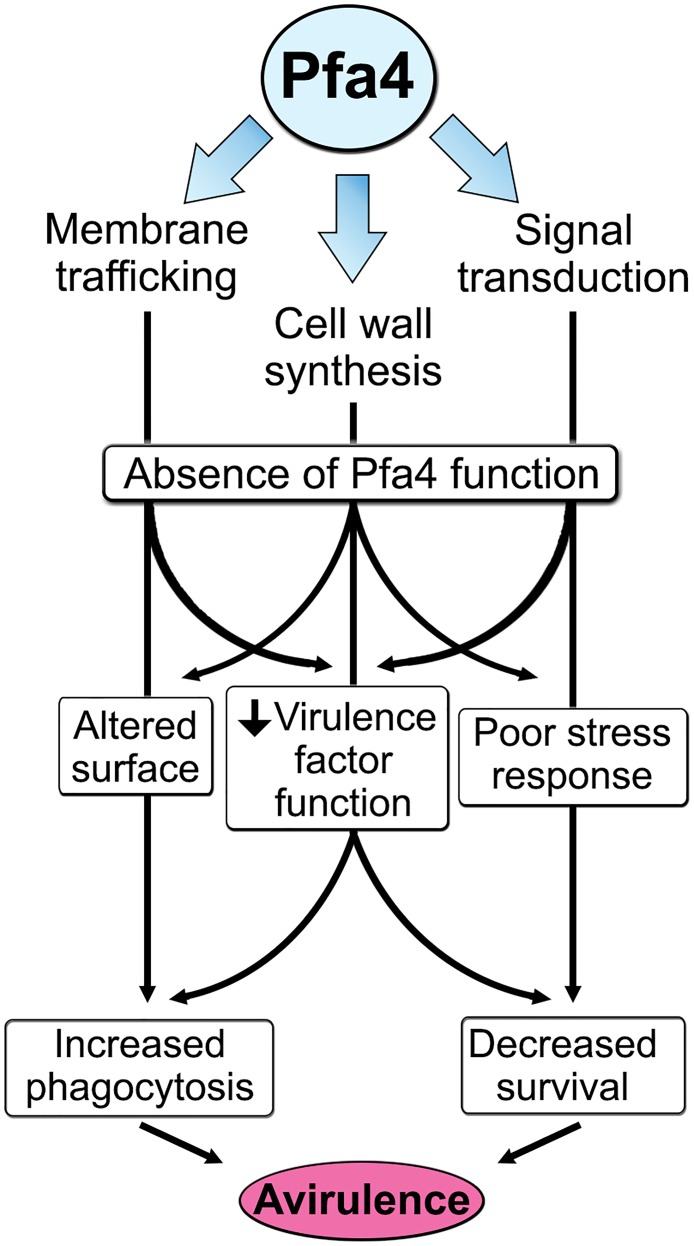
Model of Pfa4 function and relationship to morphology, stress tolerance, and virulence.

The combination of impaired cell wall synthesis and inability to appropriately respond to this condition results in weak and disorganized walls. This may impair other key virulence attributes of *C*. *neoformans*, such as the polysaccharide capsule, which associates with the cell wall. A perturbed wall, even in cells where the capsule is only slightly reduced in radius (as with *pfa4*Δ), may alter the capsule so that it cannot maintain its normal antiphagocytic role and exposes underlying wall components. This, combined with the changed wall arrangement, could explain our observation of abnormally high surface accessibility of specific cell wall components (Fig 3). These included cell wall mannoproteins [54, 55], which can interact with host phagocyte mannose receptors, and chitosan, which also interacts with macrophage receptors and induces a robust inflammatory response [56, 57]. Greater accessibility of these glycans could in turn explain the increased phagocytosis of *pfa4*Δ cells by macrophages. Once engulfed, these less robust cells, with defects in cell wall, signal transduction, and virulence factor expression, fare poorly (Fig 5A). Potentially reducing the pathogenicity even of cryptococci that remain outside of host phagocytes, we found important membrane transporters that are not correctly palmitoylated in the mutant. These include a putative carbohydrate transporter, a phosphate transporter, and *NIC1* and *SIT1*, which transport nickel and siderophore-iron complexes, respectively [58, 59]. Because these metals are limiting during infection, incorrect processing or targeting of their transporters could influence pathogenesis. Furthermore, melanin, an important virulence factor in this pathogen, is poorly retained at the cell wall (Fig 7C–7E), a phenotype also seen in *chs3*Δ cells [32] and associated with reduced virulence. Ultimately, all of these factors combine to result in avirulence of the *pfa4*Δ mutant (Fig 8).

In contrast to our findings, the initial survey of cryptococcal deletion mutants [10] categorized the strain deleted for *PFA4* (2A12) as normal in virulence. This may reflect the practical strategy used in that large-scale study, where pools of mutants were assayed, or the timing of those virulence studies. We did observe that animals infected with *pfa4*Δ initially show mild symptoms of disease, suggesting the initiation of a pathogenic process that might be interpreted as normal infectivity in short-term studies (as in ref. [10]), but that they subsequently clear the infection and recover completely. Consistent with our observations, 2A12 does show reduced virulence in recent studies of this deletion library using both an IV mouse model and invertebrate models of infection (performed at room temperature) [60]. The latter also supports our conclusion above that Pfa4’s contribution to virulence is temperature independent.

As well as demonstrating the key role of Pfa4-dependent palmitoylation in *C*. *neoformans*, our work provides valuable data sets to the community from both our screen and our palmitoylome analysis. It also bolsters a model that explains why multiple PATs have been retained during evolution despite the widespread redundancy of these enzymes: key PATs like Pfa4 may modify specific substrates that perform critical functions, in addition to sharing substrates with other PATs. This concept is supported by the recent identification of specific PATs that regulate central pathogenic processes in *Toxoplasma* and *Plasmodium* [16–18].

Our determination of the Pfa4-palmitoylome offers new insights into the role of an important regulatory lipid modification in the biology of *C*. *neoformans*. Pfa4 in *C*. *neoformans* is notable in modifying proteins that exhibit diverse modes of membrane association, including those that are otherwise predicted to be soluble or to have one or many membrane-spanning domains (Table 3). In contrast, its *S*. *cerevisiae* homolog appears to be restricted to modifying polytopic membrane proteins [28, 42]. Several cryptococcal Pfa4 substrates are also fungal-specific (e.g., the chitin synthases, vacuolar protein 8, the nickel and siderophore transporters, and the product of CNAG_02114). Finally, cryptococcal Pfa4 is unique among PATs studied to date in that it is essential for virulence in an animal model. The closest human homolog of Pfa4, DHHC6, is considerably distant in sequence homology, with the similarity restricted to the catalytic DHHC domain. These findings encourage efforts towards development of specific PAT inhibitors as novel avenues for therapeutics.

## Materials and Methods

### Strains, growth conditions, and reagents

Strains used were *C*. *neoformans* serotype A strain H99α, its derivative KN99α, and deletions in these backgrounds (see below). The cryptococcal partial deletion collection in H99α [10] was purchased from the Fungal Genetics Stock Center (University of Missouri, Kansas City, MO) and H99α *chs3*Δ was a generous gift from Jennifer Lodge (Washington University). Fungal strains were maintained at -80°C and grown at 30°C on yeast peptone dextrose (YPD) with antibiotics as appropriate (100 μg/mL of nourseothricin (clonNAT, WERNER BioAgents, Germany) or 100 μg/mL G418 (Geneticin, Life Technologies, USA)).

The human monocytic cell line THP-1 (ATCC TIB-202) was grown in THP-1 complete media (RPMI-1640 with L-glutamine supplemented with 1 mM sodium pyruvate, 0.05% 2-mercaptoethanol, 10% FBS, and 100 units/mL Penicillin- 100 μg/mL Streptomycin solution as indicated) and differentiated with phorbol 12-myristate 12-acetate (PMA, from Sigma, St. Louis, MO) as described in [9]. THP-1 cultures were split every 3–4 days (inoculum of 10^5^ cells/mL) and new batches were thawed every month.

All tissue culture plasticware and media were from BD Falcon and Sigma, fungal media components from Difco, PCR primers from Sigma, biolistic transformation reagents and materials from Bio-Rad, DH5α cells from Life Technologies, and restriction enzymes from New England Biolabs. Reagents for electron microscopy were from Ted Pella (Redding, CA) and Polysciences (Warrington, PA); antibodies for immunofluorescence were from Abcam (ab2900, anti-EEA1 rabbit polyclonal) or the Developmental Studies Hybridoma Bank (clone H4A3, University of Iowa); and antibodies for immunoblotting were from Sigma (clone HA-7 anti-HA mouse monoclonal and anti-FLAG rabbit polyclonal). Reagents for bioorthogonal labeling and click chemistry were from Sigma, except for azido-rhodamine, which was prepared as previously described [38].

### Library screening

To screen fungal mutants, THP-1 cells were seeded in 96-well plates (3.33 ×10^5^ cells/mL, 100 μL), incubated for 48 hr (37°C, 5% CO_2_) in THP-1 complete media, washed three times with 150 μL RPMI-1640, and cultured for one day in serum-free media with antibiotics. In parallel a 96-pin replicator (Nalge Nunc International, Rochester, NY) was used to inoculate strains from the *C*. *neoformans* deletion collection into a Nunc Edge—96 well microplate containing 150 μL YPD per well. The microplates were incubated at 30°C overnight on a mini-orbital shaker (BELLCO Biotechnology, Vineland, NJ), followed by transfer of a 35 μL aliquot from each well into a new 96-well flat-bottom microplate (Costar 3904). The transferred cells were washed once with PBS (pH 7.5), once in Mcllvaine’s buffer (pH 6.0), and then resuspended in 100 μL of the same buffer containing 100 μg/mL Lucifer Yellow dye (Sigma L0144). After a 30 min incubation at RT with gentle agitation the cells were collected, washed once with PBS, and opsonized (30 min, 37°C) in 100 μL 40% human serum with gentle agitation. Serum was obtained from healthy donors with informed consent under a protocol approved by the Washington University in St. Louis Institutional Review Board. Finally, the cells were washed three times with PBS, resuspended in 150 μL RPMI-1640, and 35 μL from each well was diluted into 1 mL of pre-warmed RPMI-1640 in a deep-well 96-well plate (Nunc). To initiate the assay, the medium from each well containing THP-1 was aspirated and replaced by 100 μL of the cryptococcal suspension. After a 1 hr incubation (37°C, 5% CO_2_) the plates were washed vigorously four times with 150 μL PBS using a microplate washer (ELx405TM Select CW, Biotek, Winooski, VT). The samples were then immediately fixed in 150 μL 4% formaldehyde (20 min, 4°C), washed twice with PBS, and permeabilized for 20 min at RT with 0.1% saponin in PBS (150 μL). Samples were next washed twice with PBS, stained (15 min, RT, in the dark) with 2 μg/mL DAPI and 0.25 μg/mL HCS CellMask Deep Red (Life Technologies) in PBS, washed twice more with PBS, and 100 μL of 10 mM NaN_3_ in PBS was added to each well. Plates were either imaged immediately (on an IN Cell 1000 analyzer, GE, Piscataway, NJ) or stored at 4°C for later analysis. GE INCell Investigator Developer Software was used to identify host cell and fungal borders and calculate the overlap as described in [9]. Fungal cells that overlapped >50% with host cell bodies were considered internalized, ≤50% were considered adherent, and fungal cells with no overlap were not counted. In parallel with the screening assay, an aliquot of each fungal cell suspension was pipetted into empty 96-well plates for enumeration to allow normalization of results to fungal cell number (macrophage uptake of *C*. *neoformans* is linear in the range of fungal concentrations used in these assays [9]). The results were analyzed plate-wise (to reveal any systematic errors in different plates) before normalization and calculation of values relative to wild-type.

### Fungal genome manipulation

We used the split marker method [61] to delete *PFA4* (CNAG_03981) in H99α and KN99α after amplifying NAT resistance split marker fragments from genomic DNA of strain 2A12 (*pfa4*Δ) from the Madhani deletion collection. For chromosomal complementation, we used a split marker approach to replace the NAT cassette of the mutant with wild-type genomic *PFA4* sequences in tandem with a G418 resistance cassette. For endogenous tagging of the *CHS3* gene (CNAG_05581) with mCherry, the last 1,674 bp of *CHS3* were amplified with a BamHI site replacing the STOP codon and ligated to a BamHI/AvrII-cut fragment composed of mCherry followed by an HA epitope, a STOP codon, and 445 bp of the *TRP1* terminator. The ligated fragment was cloned in front of a NAT resistance cassette and 616 bp of the *CHS3* terminator (sequences immediately following the STOP codon) were subsequently cloned after the NAT cassette. The resulting plasmid was digested with BglII/MluI to release the 5’ fragment of the split marker and with XmaI/EcoRV to release the 3’ fragment of the split marker. Transformation was by biolistics (Bio-Rad PDS-1000/He) as described in [62].

For plasmid construction, a fragment encompassing the *PFA4* coding locus and 226 bp of 3’ sequence was amplified so as to incorporate sequence that encodes 1.5X HA epitope tags in place of the first 2 codons. Fusion PCR was used to ligate this fragment to a second amplicon consisting of 900 bp of 5’ sequence (including the starting ATG) and sequence encoding 1.5X HA epitope tags, so as to reconstitute sequence encoding an N-terminal 3X HA-tagged Pfa4 sequence. This product (~3.5 kb) was cloned into ApaI/KpnI-digested pIBB103 [63] for expression and also used as template for mutagenesis of the DHHC motif into DHAS using overlapping primers containing the codon change. Plasmid transformation was as described in [63].

### Fluorescence microscopy and flow cytometry

Cells were grown overnight at 30°C in YPD (with appropriate antibiotics if needed to maintain plasmids), diluted as for phenotyping, washed in PBS, and resuspended at 10^7^/mL for staining as follows (all manipulations at RT): For LY and EoY (Sigma), cells were washed once in McIlvaines buffer, pH 6.0; resuspended in the same; and incubated for ~15 min with 250 μg/mL of the dye. For CFW (Fluorescent Brightener 28, Sigma), UV2B (Polysciences, Inc.) and Pont (Pontamine fast scarlet 4B, Bayer Corp., Robinson, PA), cells were stained in PBS with 100 μg/mL of CFW or UV2B or a 1:10,000 dilution of Pont (w/v). For ConA-FITC (Sigma), cells were stained with 30 μg/mL in Hepes-buffered saline, pH 7.0, containing 10 mM each MgCl_2_ and CaCl_2_.

For fluorescence microscopy, stained cells were washed twice, resuspended in the same volume of the corresponding buffer, mixed vigorously, spotted onto glass slides, covered, and imaged immediately on a wide field Zeiss Axioskop 2 MOT Plus with appropriate filters (DAPI for CFW and UV2B; FITC for LY, EoY, and ConA-FITC; and Texas Red for Pont). For the Chs3-mCherry strains, overnight cultures grown in YPD were washed twice with PBS, resuspended in 3 mL of PBS, and 6 μl were spotted on polylysine-coated glass slides and imaged immediately. For flow cytometry cells were washed three times, fixed in 3.7% formaldehyde/PBS (10 min; RT) or resuspended in PBS with 10 mM NaN_3_ and analyzed on an LSRII flow cytometer (Becton Dickinson, Franklin Lakes, NJ) for analysis using FlowJo software (Tree Star Inc., Ashland, OR).

### Electron microscopy (EM)

For transmission EM, overnight cultures grown in YPD were diluted 10-fold, grown to OD_600_ = 0.2 (~10^7^/mL), and washed twice in PBS. The cell pellet was resuspended in 1 mL of primary fixation mix (2.5% paraformaldehyde/2% glutaraldehyde in 100 mM cacodylate buffer, pH 7.2), incubated for 1 hr at room temperature (RT), washed in the same buffer, and post-fixed in 1% osmium tetroxide (Polysciences, Inc.) for 1 hr at RT. Samples were then rinsed in the same buffer, followed by dehydration in a graded series of ethanol and propylene oxide prior to embedding in Eponate 12 resin (Ted Pella, Inc.). Sections of 90 nm were cut with a Leica Ultracut UCT ultramicrotome (Leica Microsystems, Inc., Bannockburn, IL), stained with uranyl acetate and lead citrate, and viewed on a JEOL 1200EX transmission electron microscope (JEOL USA Inc., Peabody, MA) equipped with an AMT 8 megapixel digital camera (Advanced Microscopy Techniques, Woburn, MA).

For scanning EM, cultures were grown and fixed as above but in sodium phosphate buffer, then washed and 8.8 x 10^6^ cells (4 x 10^6^ cells/cm^2^) were added to wells of a 6-well plate containing a polylysine-coated plastic coverslip. After incubation at 4°C for 1–2 hr the coverslips were washed twice with DPBS, re-fixed in 2% paraformaldehyde, 2.5% glutaraldehyde in 0.1 M Sorensen’s sodium phosphate buffer (potassium-free, pH 7.4), and then sequentially rinsed in buffer and NanoPure Ultra-filtered deionized water. They were next post-fixed in 1% osmium tetroxide (aqueous) for 1 hr, rinsed with NanoPure Ultra-filtered deionized water, dehydrated in ethanol (30%, 50%, 70%, 80%, 90%, 3X 95%, and 3X absolute ethanol), critical point dried (Tousimis Samdri-780, Rockville, MD) via liquid carbon dioxide, mounted on aluminum stubs with double-sided adhesive carbon tabs, and sputter coated (Tousimis Samsputter-2a) with gold-palladium. Images were acquired using a Hitachi S2600 (Hitachi-hitec, Tokyo, Japan) instrument.

### Phenotyping

Strains to be tested were grown overnight in YPD, diluted to ~2 x 10^6^/ml, and grown for two doublings. The cultures were then serially diluted (10-fold) and spotted (5 μL) onto buffered (pH 6.8 with 100 mM KPO_4_ buffer) synthetic dextrose medium with 1 mg/mL calcofluor white or onto YPD with 1.2 M NaCl; 1.2 M KCl; 0.01 and 0.03% SDS; 1, 3, and 5 mM H_2_O_2_; 0.1, 0.25, 0.5, 0.75, and 1 mg/mL caffeine; 1 mg/mL Congo red (stock prepared in 70% ethanol); or 25 μg/mL Lucifer Yellow. The same plates were also prepared containing various concentrations of sorbitol (0.5, 1, or 1.5 M). Plates were incubated at 30°C and 37°C for 3–4 days. Sensitivity to lysing enzymes was tested as in [64].

### Capsule induction

Cells were grown overnight in YPD, washed twice with DMEM, and 1 mL aliquots (10^6^ cells) were pipetted into 24-well tissue culture plates (3 wells per strain) and incubated (37°C; 5% CO_2_) for 24 hr. The suspension was washed twice with deionized water (dH_2_O), resuspended in 24 μL dH_2_O, mixed with ~8 μL of India ink and visualized on a Zeiss Axioskop 2 MOT Plus microscope. At least 150 cells from each strain (50 per well) were analyzed with ImageJ (NIH) for capsule thickness ((outer capsule diameter minus cell wall diameter)/2).

### Melanin assays

For solid media assays, the cells were grown overnight in YPD medium at 30°C, diluted the next morning in 5 mL of YPD, grown to an OD_600_ of 0.2, washed twice in PBS, and adjusted to 10^7^ cells/mL in PBS. 10-fold serial dilutions were made and 5 μL of each dilution spotted on L-DOPA (1 mM) plates. The plates were incubated at 25°C, 30°C, and 37°C for 3–4 days in the dark.

For assays in liquid medium, cells of each strain were grown similarly overnight, diluted in 25 mL of YPD, and allowed to grow for 2–3 generations. At that point, the cells were washed in PBS, resuspended in 2 mL glucose-free asparagine and salts media, and the cell density was quantified. The strains were adjusted to 5 x 10^7^ cells/ml and incubated at 25°C for 18–24 hr in asparagine medium containing 1 mM L-DOPA. The cultures were spun down at 1000xg for 10 min, and photographed. To quantify the melanin in the media, the OD_405_ was measured for 100 μL aliquots of the supernatant fractions.

### Virulence assays

To assess fungal survival in macrophages, THP-1 cells grown in 12-well plates (250,000 cells per well), were washed with assay medium (RPMI + 1% FBS). In parallel, overnight fungal cultures (OD_600_ = 0.2–0.4; 1–2 x 10^7^/mL) were washed twice with PBS, resuspended (10^8^ cells/mL) in 40% human serum for opsonization (37°C; 30 min; with rotation), rewashed, resuspended in assay medium, and added to the THP-1 cells at an MOI of 0.1 or 1.0 as indicated. Plates were incubated for 1 hr, rinsed twice with 1 mL Dulbecco’s PBS (DPBS), and incubation continued for 0 hours (to measure initial association) or for the time indicated after addition of RPMI + 1%FBS. At the desired assay time points the medium was aspirated, wells were washed once with 1 mL DPBS, 1 mL of lysis buffer (0.05% SDS, 1 mM EDTA) was added, and the plate was shaken on a plate mixer for ~3 min. The resulting lysate was collected, vortexed vigorously, diluted, and spotted onto YPD media for determination of colony forming units (CFU).

To test virulence in mice, strains were cultured overnight in YPD, collected, washed in PBS, diluted to 10^6^ cells/mL in PBS, and briefly sonicated (to disperse clumps seen in *pfa4*Δ). Sonication did not adversely affect mutant viability. Aliquots (50 μL) of the suspension were used to intranasally inoculate groups of ten mice (4–6 week-old female A/Jcr mice; National Cancer Institute) and dilutions of the suspension were plated immediately after infection to confirm inocula. Animals were monitored closely and sacrificed if they lost >20% relative to peak weight or at the end of the experiment (45 days). Homogenates of lungs and brains from 3 of the surviving mice infected with *pfa4*Δ were plated to determine organ burden.

### Alk-16 labeling and click chemistry

Whole cell lysates (50 μg) were diluted with SDS buffer (4% SDS, 150 mM NaCl, 50 mM triethanolamine pH 7.4, Roche EDTA-free protease inhibitor cocktail) to 44.5 μL and then reacted with 5.5 μL freshly prepared click chemistry reaction cocktail containing azido-rhodamine (1 μL, 10 mM stock solution in DMSO), tris(2-carboxyethyl)phosphine hydrochloride (TCEP) (1 μL, 50 mM freshly prepared stock solution in deionized water), tris[(1-benzyl-1H-1,2,3-triazol-4-yl)methyl]amine (TBTA) (2.5 μL, 10 mM stock solution in DMSO/t-butanol) and CuSO_4_•5H_2_O (1 μL, 50 mM freshly prepared stock solution in deionized water)] for 1 h at room temperature. The click reactions were terminated by the addition of ice-cold methanol (1 mL). The mixtures were placed at −20°C overnight and then centrifuged at 18,000×g for 20 min at 4°C to precipitate proteins. The supernatants from the samples were discarded. The protein pellets were washed with methanol twice, allowed to air-dry for 10 min, resuspended in 35 μL of SDS lysis buffer, and diluted with 12.5 μL 4× reducing SDS-loading buffer (40% glycerol, 200 mM Tris-HCl pH 6.8, 8% SDS, 0.4% bromophenol blue) and 2.5 μL 2-mercaptoethanol. The resulting samples were heated for 5 min at 95°C and resolved on 4–20% SDS-PAGE gels (Bio-Rad). For in-gel fluorescence scanning, the gels were destained in 40% methanol, 10% acetic acid for at least 1 h, and then scanned on a GE Healthcare Typhoon 9400 variable mode imager with excitation and emission at 532 nm and 580 nm, respectively. After scanning, gels were also stained with Coomassie Brilliant Blue (Bio-Rad).

### Affinity enrichment of palmitoylated proteins and mass spectrometry

For affinity purification of alk-16-modified proteins, 2 mg of cell lysates labeled with alk-16 were subjected to Cu(I)-catalyzed click reaction as described above, except that azido-biotin was substituted for azido-rhodamine. Methanol-precipitated and washed protein pellets were resuspended in 200 μL of 4% SDS buffer (50 mM TEA, 150 mM NaCl, pH 7.4). Equal amounts of protein for each sample were diluted 1/4 by volume with 50 mM TEA buffer (150 mM NaCl, pH 7.4). 60 μl prewashed streptavidin agarose beads (Invitrogen) were added to each sample and the protein and bead mixtures were incubated for 1 h at room temperature on a nutating mixer. The beads were then washed once with PBS and 0.2% (w/v) SDS, three times with PBS and twice with 250 mM ammonium bicarbonate (ABC). Beads were resuspended in 500 μl 8 M urea, reduced with 10 mM DTT for 30 min, and then alkylated with 50 mM iodoacetamide in the dark for another 30 min. Finally, the beads were washed with 25 mM ammonium bicarbonate three times and digested with 0.5 μg of trypsin at 37°C overnight. The supernatant of each sample was collected, dried, and solubilized in 5% acetonitrile/1% formic acid for LC-MS analysis.

LC-MS analysis was performed with a Dionex 3000 nano-HPLC coupled to an Orbitrap XL mass spectrometer (ThermoFisher). Peptide samples were pressure-loaded onto a home-made C18 reverse-phase column (75 μm diameter, 15 cm length). A 180-minute gradient increasing from 95% buffer A (HPLC grade water with 0.1% formic acid) and 5% buffer B (HPLC grade acetonitrile with 0.1% formic acid) to 75% buffer B in 133 minutes was used at 200 nL/min. The Orbitrap XL was operated in top-8-CID-mode with MS spectra measured at a resolution of 60,000@m/z 400. One full MS scan (300–2000 MW) was followed by three data-dependent scans of the nth most intense ions with dynamic exclusion enabled. Acquired tandem MS spectra were extracted using ProteomeDiscoverer v.1.4.0.288 (Thermo, Bremen, Germany) and queried against the Uniprot complete *Cryptococcus neoformans var*. *grubii* H99 proteome (UP000010091) database concatenated with common known contaminants using MASCOT v.2.3.02 (Matrixscience, London, UK). Peptides fulfilling a Percolator calculated 1% false discovery rate (FDR) threshold were reported. The abundance of an identified protein was calculated based on the average area of its three most abundant peptides. For a protein to be considered a Pfa4-specific substrate, it had to be at least five-fold more abundant in the wild-type sample compared to the *pfa4*Δ sample as measured by protein abundance in both of the two independent experiments and be identified with at least two unique peptides.

## Supporting Information

S1 Fig Effects of labeling method and cell viability on fungal uptake. (A and B) High phagocytosis of *pfa4*Δ cells is independent of labeling method. THP-1 uptake assays were performed as in Fig 1, but the fungal cells were either stained for 30 min with 10 μM DFFDA-SE (a cell permeant, non-fluorescent compound that is retained only in live cells, where it becomes highly fluorescent; Invitrogen) or were left unstained (B) for subsequent labeling with anti-capsule antibody 3C2 (as in ref. [65]; antibody generously provided by Tom Kozel). *, P < 0.001 (Mann Whitney t test) comparing mutants with respective parent strains. (C) THP-1 uptake assays performed as in Fig 1, with live H99, or H99 killed by incubation at 70 or 100°C or with ethanol or azide.
(TIF)

S2 Fig Dynamics and intracellular location of fungal cells. (A) Fungal cell localization over time, with cells classified as external to THP-1 cells (Adherent), internalized but with no marker association (Unlabeled), or internalized and associated with either an early endosome marker (EEA1+) or late endosome/lysosomal (LAMP-1+) markers. Average ± SEM from manual counts of ≥100 cells from three independent studies are shown. *, P < 0.05 (Student’s t-test) for mutant versus wild-type; color of * indicates category being compared. (B) Representative images of THP-1 cells incubated with the indicated strains (stained with LY, green) for 1 hr, washed to remove non-associated fungal cells, and labeled with Lysotracker Red (red) for an additional hour prior to imaging. The DIC and merged images are displayed at right. Note that fungi that were heat-killed (HK) prior to staining and assay appear as solidly stained shapes rather than silhouettes. (C) Quantification of the staining pattern of the *Cryptococcus*-containing phagosomes (CCPs) from (B). Shown are the averages ± SD from two independent assays, counting ≥ 100 CCPs for each strain per experiment. Blue, unstained phagosomes; pink, phagosomes with a rim of Lysotracker Red around the yeast (shown as yellow in merge at right), suggesting viable fungi in an acidified compartment; green, phagosomes completely stained with Lysotracker Red, suggesting dead fungi in an acidified compartment. Examples of each category are shown at right.
(TIF)

S3 Fig DIC images of unstained *C*. *neoformans*. Representative images of the indicated strains grown at 30°C and visualized by DIC. Scale bars, 5 μm on main panels, 2 μm on magnified regions. For the *pfa4*Δ cell panels, three images (corresponding to the expanded region) taken 1 μm apart in a z-stack are shown, to better depict the surface topology.
(TIF)

S4 Fig Images of *C*. *neoformans* stained for flow cytometry. Representative images of the indicated strains grown at 30°C and stained with Lucifer Yellow (LY), pontamine (Pont), calcofluor white (CFW), eosin Y (EoY), and concanavalin A conjugated to FITC (ConA). Scale bar, 5 μm.
(TIF)

S5 Fig Cell wall-related phenotypes of wild-type, *pfa*4Δ, and Pfa4 active site mutant strains. (A) *pfa*4Δ cells grow slowly but maintain viability during growth in mammalian tissue culture medium. Overnight cultures grown at 30°C in YPD were washed and diluted to 10^5^ cells/mL in prewarmed RPMI in tissue culture flasks. The flasks were incubated at 37°C with 5% CO_2_, and aliquots were taken for cell counting and CFU determination. The graph is representative of cell counts from three independent experiments. CFUs at all time points showed viable cryptococci from all strains, although the viability of the *pfa*4Δ cells was typically 40–60% compared to 70% or above for wild-type. (B) 5 μL of 10-fold serial dilutions were spotted on plates containing the indicated stressors and incubated for 3–4 days. The *pfa4*Δ mutant is sensitive to a variety of cell stresses, but all phenotypes are rescued by complementation with *PFA4*. (C) Cell lysis over time during treatment with lysing enzymes from *Trichoderma harzianum*, as described in the Materials and Methods. (D) Alignment of the canonical consensus sequence of a DHHC-CRD domain [11] with the corresponding domain in Pfa4; underlined amino acids (DHHC) are the catalytic residues and arrows indicate the mutations made to generate *pfa4* ^AS^. (E) 10-fold serial dilutions were spotted as in (B). The mutant *pfa4* ^AS^ construct and vector alone do not complement the *pfa4* deletion, although the wild-type *PFA4* construct does.
(TIF)

S6 Fig Hyphal filamentation in *pfa4* strains set. Wild-type (KN99) and *pfa4*Δ cells of both mating types were crossed in a 2 x 2 matrix on V8 mating media (see [29] for details). The plates were incubated in the dark for 14 days. Lack of mating filaments in the *pfa4*Δ crosses (lower right) indicates a defective mating pathway. The scale bars on each panel represent 100 pixels. All pictures were taken at the same magnification, but the scale is different for the wild-type cross (top left; depicted by a white scale bar) to capture the more abundant and longer filaments.
(TIF)

S1 Table *C*. *neoformans* mutants showing altered interactions with macrophages. (PDF)

S1 File Pfa4-specific substrates (see text and Materials and Methods). (XLSX)

S1 Video Z-stack compiled into a video of THP-1 cells (blue) after 1 hr exposure to *C*. *neoformans* wild-type cells (shown in magenta) at an MOI of 5. (AVI)

S2 Video Z-stack compilled into a video of THP-1 cells (blue) after 1 hr exposure to *C*. *neoformans pfa4*Δ cells (shown in magenta) at an MOI of 5. (AVI)
