# Supplementary figures and images for: A Single Protein S-acyl Transferase Acts through Diverse Substrates to Determine Cryptococcal Morphology, Stress Tolerance, and Pathogenic Outcome
Source: PLoS Pathog. 2015 May 13;11(5):e1004908. doi: 10.1371/journal.ppat.1004908 (PMC4430228; doi:10.1371/journal.ppat.1004908)

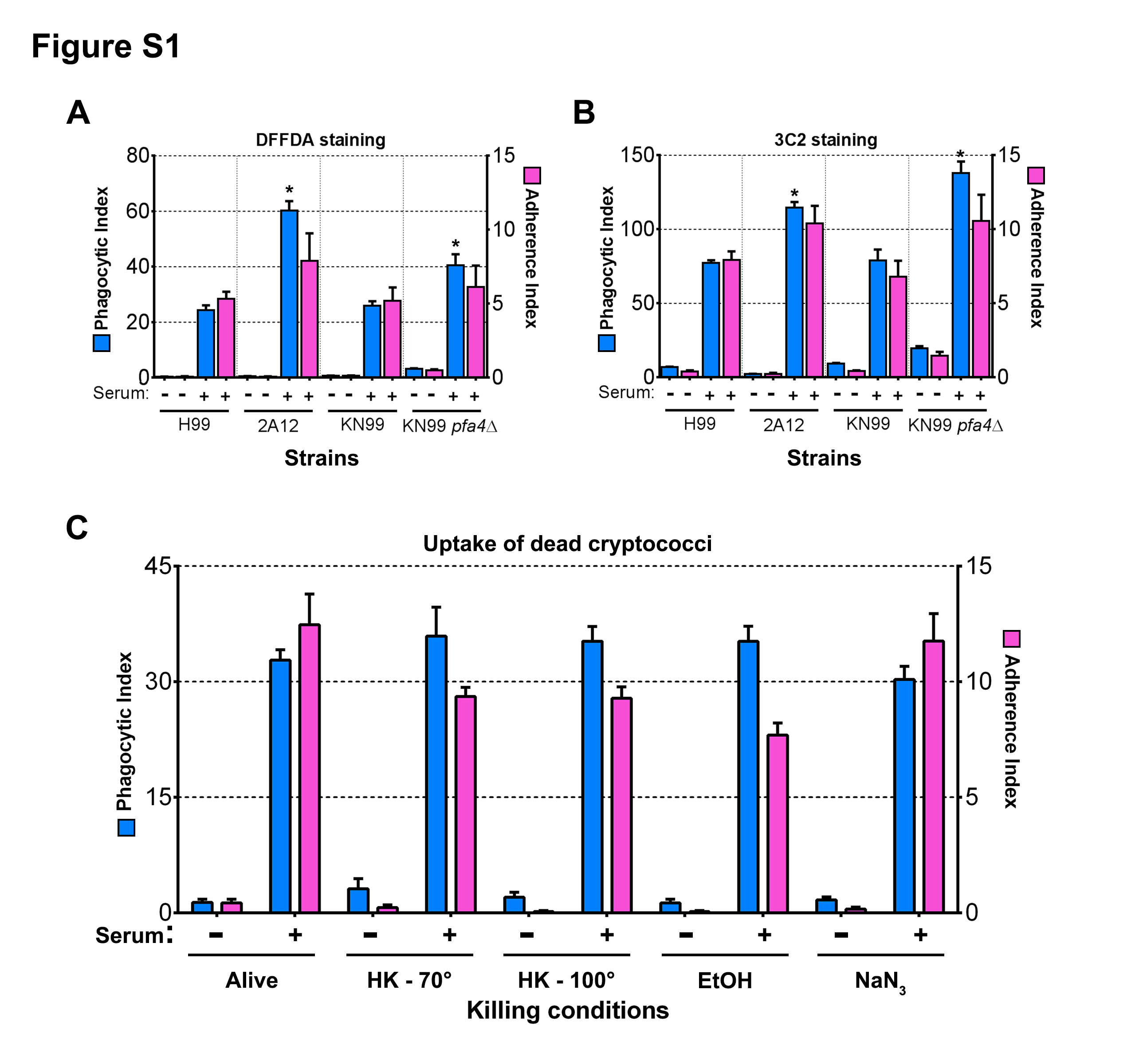

Supplement: S1 Fig — (A and B) High phagocytosis of pfa4Δ cells is independent of labeling method. THP-1 uptake assays were performed as in Fig 1, but the fungal cells were either stained for 30 min with 10 μM DFFDA-SE (a cell permeant, non-fluorescent compound that is retained only in live cells, where it becomes highly fluorescent; Invitrogen) or were left unstained (B) for subsequent labeling with anti-capsule antibody 3C2 (as in ref. [65]; antibody generously provided by Tom Kozel). *, P < 0.001 (Mann Whitney t test) comparing mutants with respective parent strains. (C) THP-1 uptake assays performed as in Fig 1, with live H99, or H99 killed by incubation at 70 or 100°C or with ethanol or azide. (TIF) [file ppat.1004908.s001.tif]

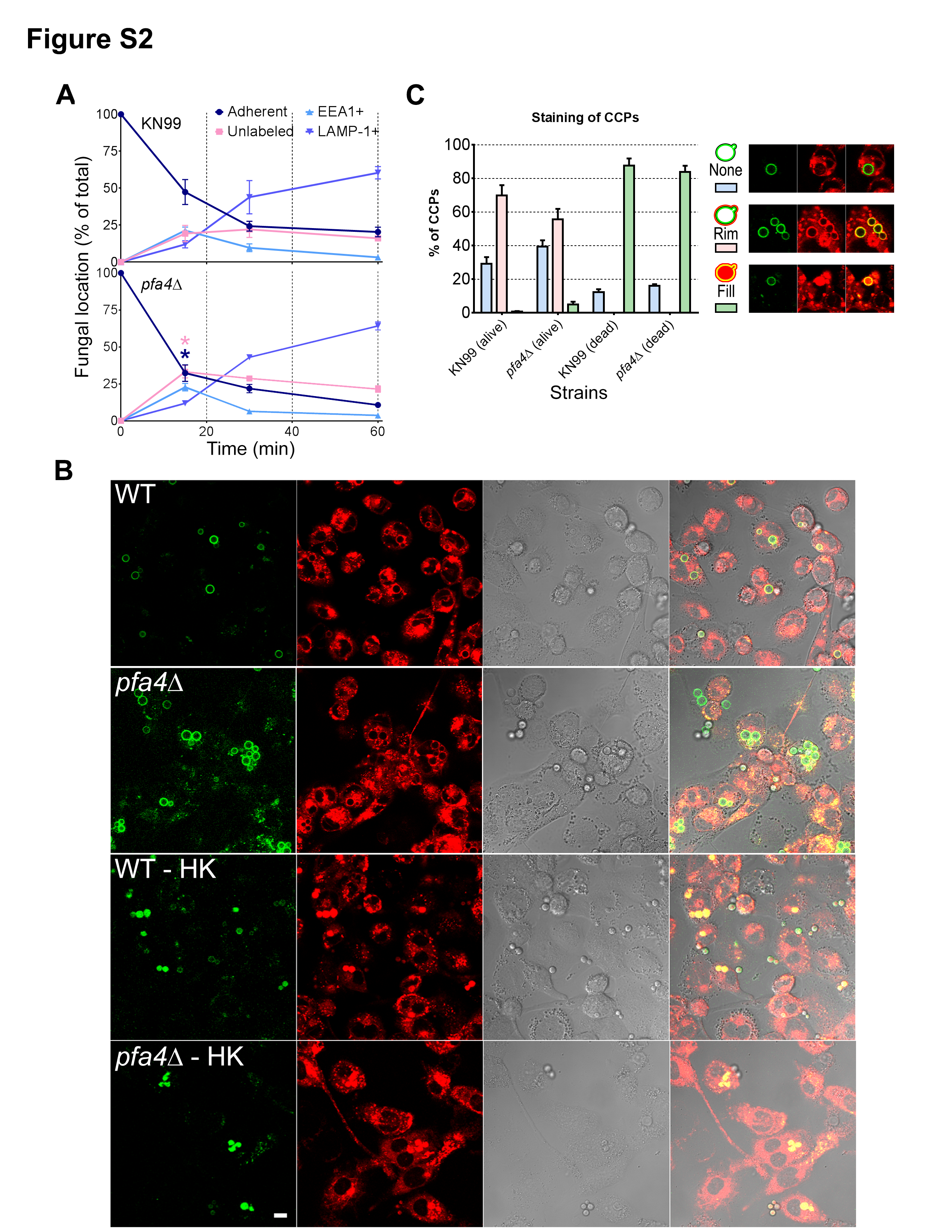

Supplement: S2 Fig — (A) Fungal cell localization over time, with cells classified as external to THP-1 cells (Adherent), internalized but with no marker association (Unlabeled), or internalized and associated with either an early endosome marker (EEA1+) or late endosome/lysosomal (LAMP-1+) markers. Average ± SEM from manual counts of ≥100 cells from three independent studies are shown. *, P < 0.05 (Student’s t-test) for mutant versus wild-type; color of * indicates category being compared. (B) Representative images of THP-1 cells incubated with the indicated strains (stained with LY, green) for 1 hr, washed to remove non-associated fungal cells, and labeled with Lysotracker Red (red) for an additional hour prior to imaging. The DIC and merged images are displayed at right. Note that fungi that were heat-killed (HK) prior to staining and assay appear as solidly stained shapes rather than silhouettes. (C) Quantification of the staining pattern of the Cryptococcus-containing phagosomes (CCPs) from (B). Shown are the averages ± SD from two independent assays, counting ≥ 100 CCPs for each strain per experiment. Blue, unstained phagosomes; pink, phagosomes with a rim of Lysotracker Red around the yeast (shown as yellow in merge at right), suggesting viable fungi in an acidified compartment; green, phagosomes completely stained with Lysotracker Red, suggesting dead fungi in an acidified compartment. Examples of each category are shown at right. (TIF) [file ppat.1004908.s002.tif]

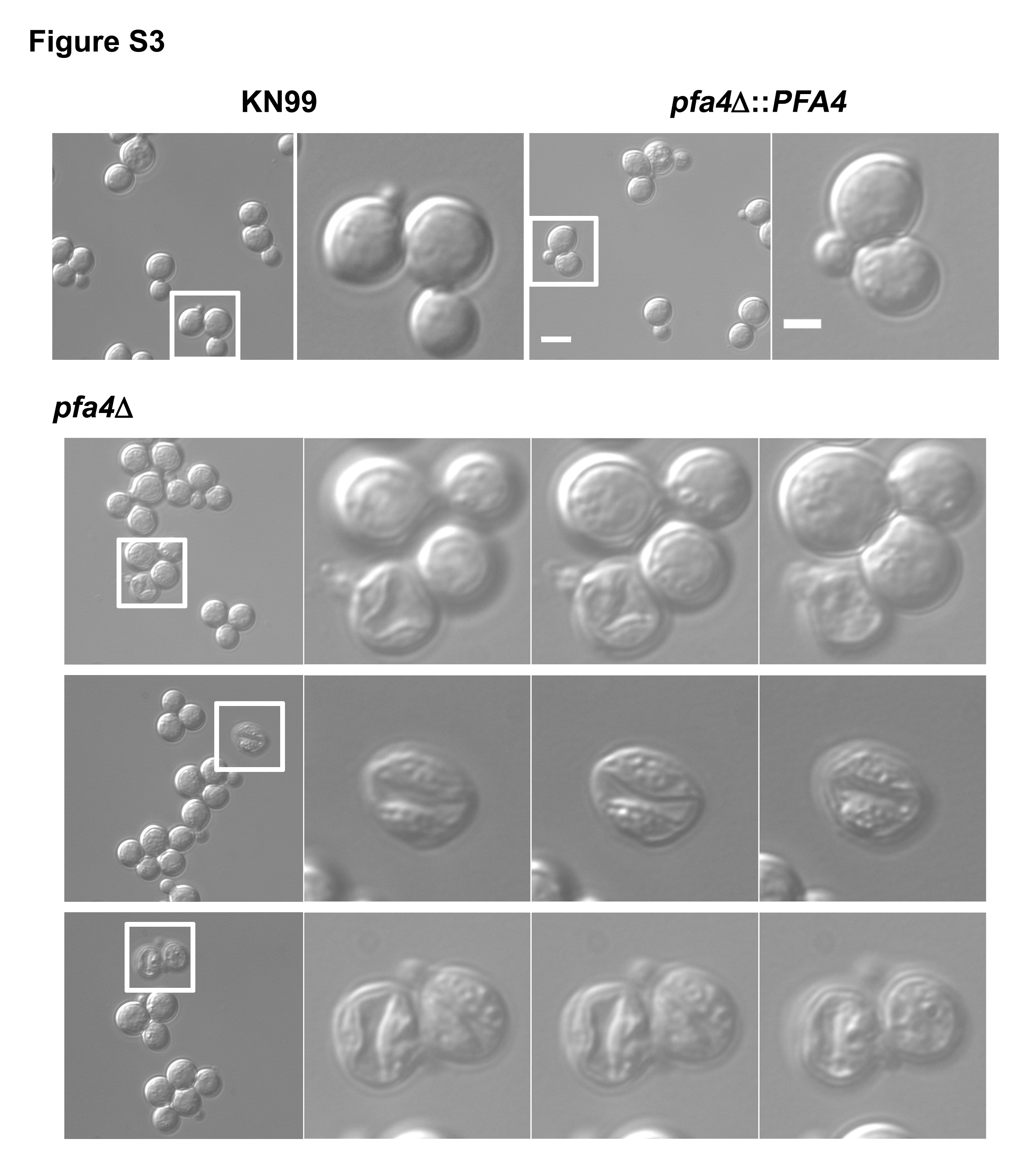

Supplement: S3 Fig — Representative images of the indicated strains grown at 30°C and visualized by DIC. Scale bars, 5 μm on main panels, 2 μm on magnified regions. For the pfa4Δ cell panels, three images (corresponding to the expanded region) taken 1 μm apart in a z-stack are shown, to better depict the surface topology. (TIF) [file ppat.1004908.s003.tif]

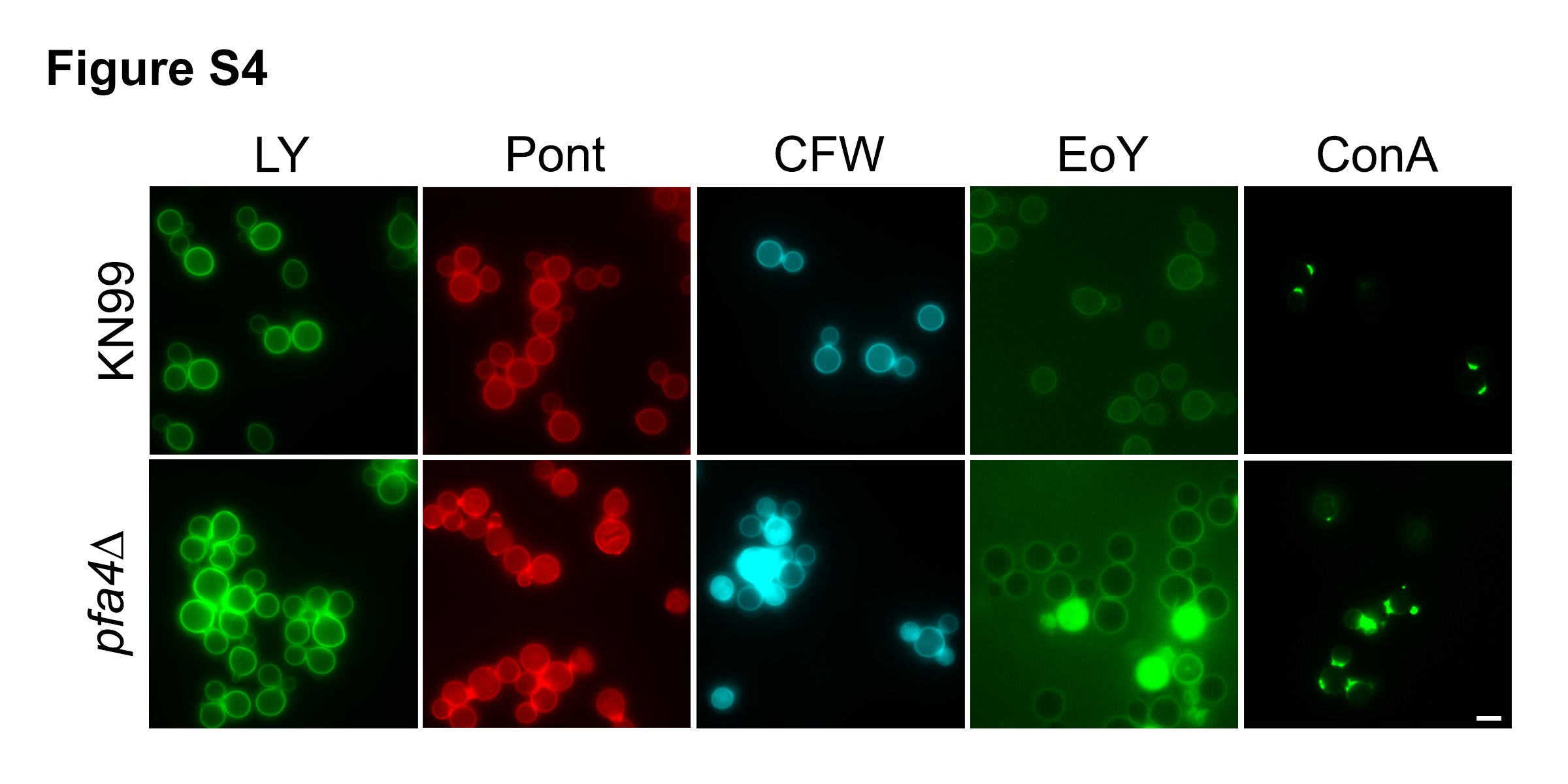

Supplement: S4 Fig — Representative images of the indicated strains grown at 30°C and stained with Lucifer Yellow (LY), pontamine (Pont), calcofluor white (CFW), eosin Y (EoY), and concanavalin A conjugated to FITC (ConA). Scale bar, 5 μm. (TIF) [file ppat.1004908.s004.tif]

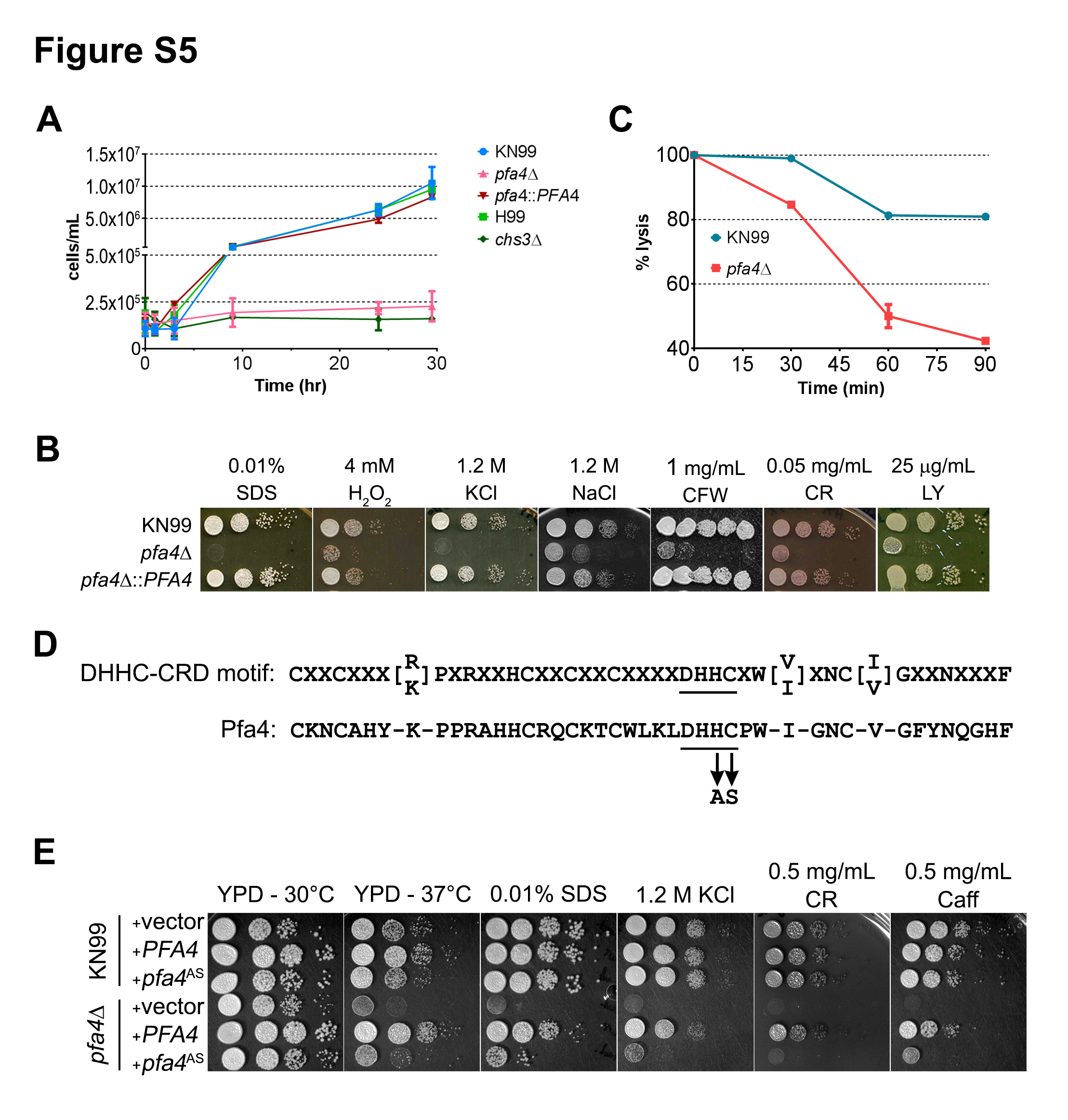

Supplement: S5 Fig — (A) pfa4Δ cells grow slowly but maintain viability during growth in mammalian tissue culture medium. Overnight cultures grown at 30°C in YPD were washed and diluted to 105 cells/mL in prewarmed RPMI in tissue culture flasks. The flasks were incubated at 37°C with 5% CO2, and aliquots were taken for cell counting and CFU determination. The graph is representative of cell counts from three independent experiments. CFUs at all time points showed viable cryptococci from all strains, although the viability of the pfa4Δ cells was typically 40–60% compared to 70% or above for wild-type. (B) 5 μL of 10-fold serial dilutions were spotted on plates containing the indicated stressors and incubated for 3–4 days. The pfa4Δ mutant is sensitive to a variety of cell stresses, but all phenotypes are rescued by complementation with PFA4. (C) Cell lysis over time during treatment with lysing enzymes from Trichoderma harzianum, as described in the Materials and Methods. (D) Alignment of the canonical consensus sequence of a DHHC-CRD domain [11] with the corresponding domain in Pfa4; underlined amino acids (DHHC) are the catalytic residues and arrows indicate the mutations made to generate pfa4 AS. (E) 10-fold serial dilutions were spotted as in (B). The mutant pfa4 AS construct and vector alone do not complement the pfa4 deletion, although the wild-type PFA4 construct does. (TIF) [file ppat.1004908.s005.tif]

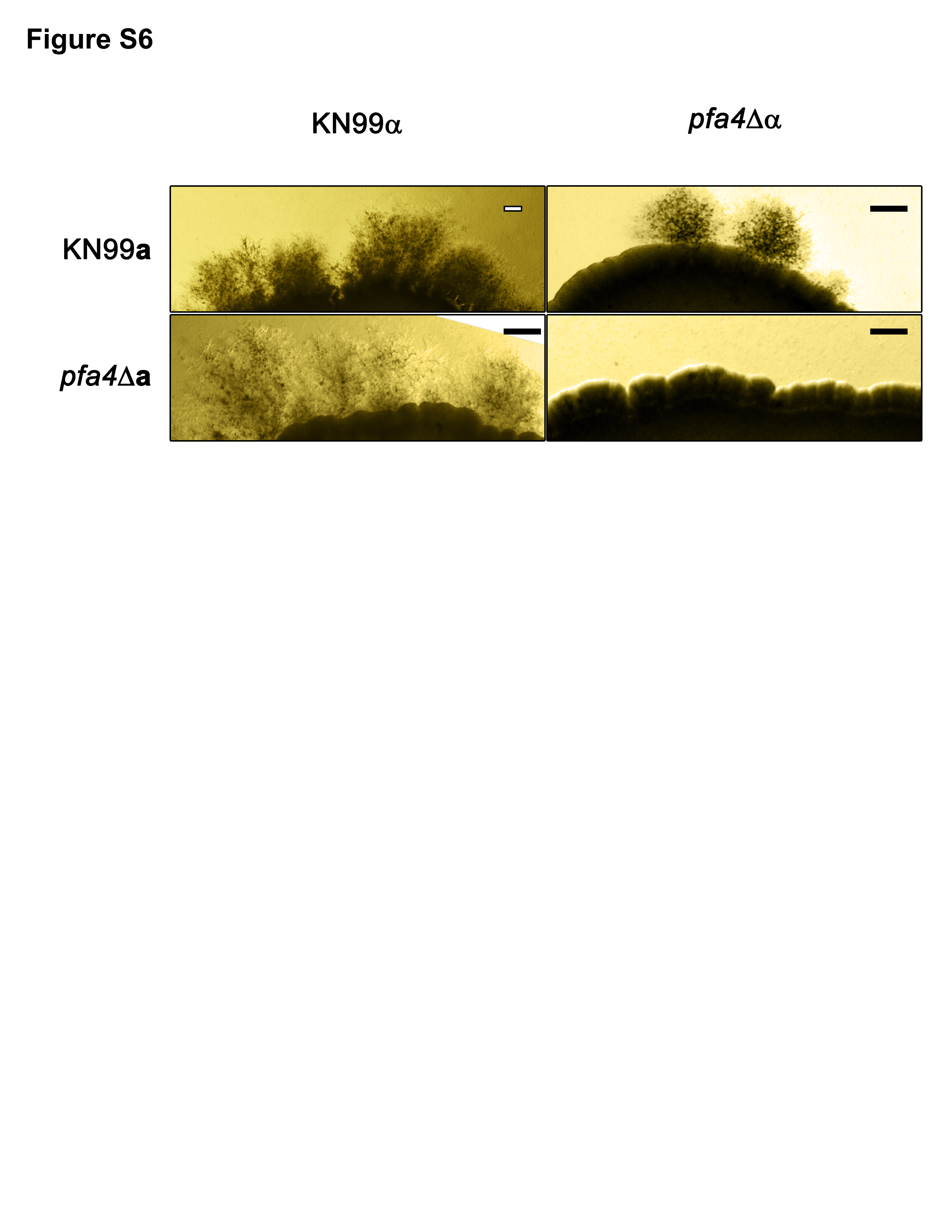

Supplement: S6 Fig — Wild-type (KN99) and pfa4Δ cells of both mating types were crossed in a 2 x 2 matrix on V8 mating media (see [29] for details). The plates were incubated in the dark for 14 days. Lack of mating filaments in the pfa4Δ crosses (lower right) indicates a defective mating pathway. The scale bars on each panel represent 100 pixels. All pictures were taken at the same magnification, but the scale is different for the wild-type cross (top left; depicted by a white scale bar) to capture the more abundant and longer filaments. (TIF) [file ppat.1004908.s006.tif]
