# Supplementary material for: A Single Protein S-acyl Transferase Acts through Diverse Substrates to Determine Cryptococcal Morphology, Stress Tolerance, and Pathogenic Outcome
Source: PLoS Pathog. 2015 May 13;11(5):e1004908. doi: 10.1371/journal.ppat.1004908 (PMC4430228; doi:10.1371/journal.ppat.1004908)
Supplement: S1 Table — (PDF) [file ppat.1004908.s007.pdf]

**Table S1. *C. neoformans* mutants showing altered interactions with macrophages[a]**

A. Increased uptake mutants

| Index[b] | Library well[c] | Gene ID | Gene name[d] | Description and/or functional domains | Reference |
|---|---|---|---|---|---|
| 2.51 | 4C6 | CNAG_01172 | *PBX1* | Parallel beta-helix repeat protein; surface glycan synthesis/remodeling | Kumar et al., 2014; Liu et al., 2007 |
| 2.42 | 8E6 | CNAG_02797 | *CPL1* | Putative secreted protein; capsule synthesis and/or assembly | Cai et al., 2014; Liu et al., 2008 |
| 2.25 | 2G9 | CNAG_05431 | *RIM101* | Transcription factor; regulation of cell wall assembly in response to pH | O'Meara et al., 2013; O'Meara et al., 2014 |
| 2.11 | 11A5 | CNAG_04514 | *MPK1* | MAP kinase; cell integrity signaling and metabolite resistance | Kraus et al., 2003 |
| 2.10 | 9H11 | CNAG_03018 | ***ASG101*** | Zinc finger transcription factor; homologous to *S. cerevisiae ASG1* | This study |
| 1.90 | 2E9 | CNAG_00396 | *PKA1* | cAMP dependent protein kinase; mating and virulence signaling | Choi et al., 2012; D'Souza et al., 2001 |
| 1.77 | 12B2 | CNAG_01551 | *GAT201* | Transcription factor; regulation of anti-phagocytic mechanisms | Chun et al., 2011; Liu et al., 2008 |
| 1.72 | 1A9 | CNAG_06086 | *CDK8* | Cyclin-dependent protein kinase 8 | FungiDB |
| 1.50 | 10D3 | CNAG_04863 | *VPS25* | Component of the ESCRT complex; protein sorting/degradation | Chun and Madhani, 2010; Liu et al., 2008 |
| 1.43 | 8F11 | CNAG_03188 | *SET202* | Histone-lysine N-methyltransferase | Liu et al., 2008 |
| 1.37 | 7A3 | CNAG_01483 | ***HPI1*** | Ring/zinc finger; similar to E3 ubiquitin ligases | This study |
| 1.34 | 13H5 | CNAG_03409 | *SKN7* | Two-component system response regulator; regulates stress responses | Bahn et al., 2006; Coenjaerts et al., 2006 |
| 1.33 | 9D8 | CNAG_04158 | ***HIR1*** | Nucleosome assembly complex protein, involved in histone gene transcription | This study |
| 1.32 | 1C12 | CNAG_04118 | ***CTK1*** | CMGC/CDK/CRK7 protein kinase | This study |
| 1.29 | 2H1 | CNAG_00375 | *GCN5* | SAGA complex histone acetyltransferase | O'Meara et al., 2010 |
| 1.28 | 3H6 | CNAG_07253 | ***HPI2*** | Cupin-like domain, conserved in *Cryptococcus*; no homologues in *S. cerevisiae* | This study |
| 1.23 | 4A7 | CNAG_00455 | *UBP16* | ubiquitin carboxyl-terminal hydrolase | FungiDB |
| 1.18 | 4D3 | CNAG_07315 | *LIV6* | CDA08-related protein (T-cell immuno-modulatory protein homolog); possibly involved in endosome function | Brown and Madhani, 2012; Liu et al., 2008 |
| 1.18 | 7H1 | CNAG_03202 | *CAC1* | Adenylate cyclase | Alspaugh et al., 2002 |
| 1.17 | 1E2 | CNAG_06762 | *GAT204* | GATA transcription factor; capsule-independent antiphagocytic protein | Chun et al., 2011; Haynes et al., 2011 |
| 1.17 | 12C1 | CNAG_00110 | ***HPI3*** | Hypothetical protein; no homolog in *S. cerevisiae* | This study |
| 1.14 | 12A11 | CNAG_05590 | *TCO2* | Two-component-like sensor kinase | Bahn et al., 2006 |
| 1.14 | 2A12 | CNAG_03981 | ***PFA4*** | DHHC protein palmitoyltransferase | This study |
| 1.14 | 3E10 | CNAG_05787 | ***HPI4*** | Similar to symplekin protein | This study |
| 1.14 | 3G5 | CNAG_00367 | *DYN5* | Dynactin Arp1 p25 subunit | FungiDB |

B. Decreased uptake mutants

| Index[b] | Library well[c] | Gene ID | Gene name[d] | Description and/or functional domains | Reference |
|---|---|---|---|---|---|
| -4.05 | 4H8 | CNAG_01964 | *OPT1* | Proton-coupled oligopeptide transporter | FungiDB |
| -2.71 | 4C12 | CNAG_01640 | ***CSF1*** | Hypothetical protein; homologous to *S. cerevisiae CSF1* | This study |

| | | | | | |
|---|---|---|---|---|---|
| -2.56 | 9B5 | CNAG_06759 | ***LPI1*** | Dehydrogenase | This study |
| -2.47 | 5G8 | CNAG_07351 | ***LPI2*** | Hypothetical protein; no homologs in *S. cerevisiae* | This study |
| -2.41 | 4H9 | CNAG_06370 | *BAT2* | Branched-chain-amino-acid aminotransferase | FungiDB |
| -2.21 | 12D6 | CNAG_02580 | ***LPI3*** | Hypothetical protein; no homologs in *S. cerevisiae* | This study |
| -2.01 | 9E4 | CNAG_01262 | *GPB1* | G-protein β-subunit involved in pheromone sensing and mating | Wang et al., 2000 |
| -2.01 | 9A12 | CNAG_06074 | ***LPI4*** | Cytoplasmic protein of unknown function | This study |
| -2.00 | 10H11 | CNAG_00414 | ***MAK32*** | Hypothetical protein; homologous to *S. cerevisiae MAK32* | This study |
| -1.96 | 1F6 | CNAG_07534 | ***TRS130*** | Hypothetical protein; homologous to *S. cerevisiae TRS130* | This study |
| -1.83 | 10H1 | CNAG_03912 | ***LPI5*** | Transporter of the major facilitator superfamily | This study |
| -1.83 | 9H5 | CNAG_04461 | *HFM1* | ATP-dependent DNA helicase | FungiDB |
| -1.79 | 2E8 | CNAG_04256 | ***LPI6*** | Hypothetical protein; no homologs in *S. cerevisiae* | This study |
| -1.78 | 12F2 | CNAG_07580 | ***LPI7*** | CAMK protein kinase; domains typical of serine/threonine kinases but also methyltransferases and DNA binding | This study |
| -1.77 | 7G5 | CNAG_06986 | ***LPI8*** | Sugar transporter | This study |
| -1.71 | 9G10 | CNAG_05411 | ***LPI9*** | Endoglucanase | This study |
| -1.63 | 10H4 | CNAG_04546 | ***LPI10*** | Multidrug transporter | This study |
| -1.63 | 10E9 | CNAG_00745 | *HRK1* | Serine/threonine-protein kinase | Kim et al., 2011 |
| -1.62 | 12D3 | CNAG_05077 | ***LPI11*** | Glycosyl hydrolase | This study |
| -1.59 | 4F3 | CNAG_07180 | *SIR2* | NAD-dependent histone deacetylase | FungiDB |
| -1.58 | 7H6 | CNAG_02581 | *CAS33* | Capsular associated protein; involved in modification of GXM polysaccharide | Moyrand et al., 2004 |
| -1.57 | 9D5 | CNAG_02254 | ***LPI12*** | Transporter of the major facilitator superfamily; similar to quinate permeases | This study |
| -1.53 | 6H4 | CNAG_00387 | ***LPI13*** | Hypothetical protein; domains typical of GTPase activating proteins | This study |
| -1.51 | 4A12 | CNAG_04681 | ***LPI14*** | Hypothetical transmembrane protein; no homologs in *S. cerevisiae* | This study |
| -1.47 | 9D2 | CNAG_00699 | ***LPI15*** | Membrane protein; domains typical of WSC proteins, polycystin, and fungal exoglucanase | This study |
| -1.44 | 6G4 | CNAG_05468 | ***APN1*** | Apurinic/apyrimidinic endonuclease; homologous to *S. cerevisiae APN1* | This study |
| -1.44 | 7D9 | CNAG_00353 | ***LPI16*** | 3-oxo-5-alpha-steroid 4-dehydrogenase (steroid reductase) | This study |
| -1.44 | 1D5 | CNAG_07422 | *DHA1* | Glycoprotein that elicits a delayed-type hypersensitivity response in mice | Mandel et al., 2000 |
| -1.43 | 6G8 | CNAG_02941 | ***LPI17*** | Hypothetical protein; no homologs in *S. cerevisiae* | This study |
| -1.42 | 1E6 | CNAG_06290 | *SNF3/ HXT2*[e] | Low-affinity glucose (hexose) transporter | Liu et al., 2013 |

[a] All 56 mutants identified in the screen, listed by increased (A) or decreased (B) phagocytic index.
[b] Value shown is the average (on a binary log scale) of the adjusted uptake of each strain from three independent screens.
[c] Location of the strain in the deletion collection (see Liu et al., 2008).
[d] Bold indicates new names given to uncharacterized genes either based on homology to *S. cerevisiae* per nomenclature guidelines (see Inglis et al., 2014) or, for genes with no homology to *S. cerevisiae*, based on phenotype: *HPI* or *LPI* mutants, for High or Low Phagocytic Index.
[e] Gene CNAG_06290 appears in fungal databases as *SNF3* but was published as *HXT2* (Liu et al., 2013).

Alspaugh, J.A., Pukkila-Worley, R., Harashima, T., Cavallo, L.M., Funnell, D., Cox, G.M., Perfect, J.R., Kronstad, J.W., and Heitman, J. (2002). Adenylyl cyclase functions downstream of the Galpha protein Gpa1 and controls mating and pathogenicity of *Cryptococcus neoformans*. Eukaryotic cell *1*, 75-84.
Bahn, Y.S., Kojima, K., Cox, G.M., and Heitman, J. (2006). A unique fungal two-component system regulates stress responses, drug sensitivity, sexual development, and virulence of *Cryptococcus neoformans*. Molecular biology of the cell *17*, 3122-3135.
Brown, J.C., and Madhani, H.D. (2012). Approaching the functional annotation of fungal virulence factors using cross-species genetic interaction profiling. PLoS genetics *8*, e1003168.
Cai, J.P., Liu, L.L., To, K.K., Lau, C., Woo, P., Lau, S., Guo, Y.H., Ngan, A.H., Che, X.Y., and Yuen, K.Y. (2014). Characterization of the antigenicity of Cpl1 protein, a surface protein of *Cryptococcus neoformans* var. *neoformans*. Mycologia.
Choi, J., Vogl, A.W., and Kronstad, J.W. (2012). Regulated expression of cyclic AMP-dependent protein kinase A reveals an influence on cell size and the secretion of virulence factors in *Cryptococcus neoformans*. Molecular microbiology *85*, 700-715.
Chun, C.D., Brown, J.C., and Madhani, H.D. (2011). A major role for capsule-independent phagocytosis-inhibitory mechanisms in mammalian infection by *Cryptococcus neoformans*. Cell host & microbe *9*, 243-251.
Chun, C.D., and Madhani, H.D. (2010). Ctr2 links copper homeostasis to polysaccharide capsule formation and phagocytosis inhibition in the human fungal pathogen *Cryptococcus neoformans*. PloS one *5*.
Coenjaerts, F.E., Hoepelman, A.I., Scharringa, J., Aarts, M., Ellerbroek, P.M., Bevaart, L., Van Strijp, J.A., and Janbon, G. (2006). The Skn7 response regulator of *Cryptococcus neoformans* is involved in oxidative stress signalling and augments intracellular survival in endothelium. FEMS yeast research *6*, 652-661.
D'Souza, C.A., Alspaugh, J.A., Yue, C., Harashima, T., Cox, G.M., Perfect, J.R., and Heitman, J. (2001). Cyclic AMP-dependent protein kinase controls virulence of the fungal pathogen *Cryptococcus neoformans*. Molecular and cellular biology *21*, 3179-3191.
Haynes, B.C., Skowyra, M.L., Spencer, S.J., Gish, S.R., Williams, M., Held, E.P., Brent, M.R., and Doering, T.L. (2011). Toward an integrated model of capsule regulation in *Cryptococcus neoformans*. PLoS pathogens *7*, e1002411.
Kim, S.Y., Ko, Y.J., Jung, K.W., Strain, A., Nielsen, K., and Bahn, Y.S. (2011). Hrk1 plays both Hog1-dependent and -independent roles in controlling stress response and antifungal drug resistance in *Cryptococcus neoformans*. PloS one *6*, e18769.
Kraus, P.R., Fox, D.S., Cox, G.M., and Heitman, J. (2003). The *Cryptococcus* neoformans MAP kinase Mpk1 regulates cell integrity in response to antifungal drugs and loss of calcineurin function. Molecular microbiology *48*, 1377-1387.
Kumar, P., Heiss, C., Santiago-Tirado, F.H., Black, I., Azadi, P., and Doering, T.L. (2014). Pbx proteins in *Cryptococcus* neoformans cell wall remodeling and capsule assembly. Eukaryotic cell *13*, 560-571.
Liu, O.W., Chun, C.D., Chow, E.D., Chen, C., Madhani, H.D., and Noble, S.M. (2008). Systematic genetic analysis of virulence in the human fungal pathogen *Cryptococcus neoformans*. Cell *135*, 174-188.
Liu, O.W., Kelly, M.J., Chow, E.D., and Madhani, H.D. (2007). Parallel beta-helix proteins required for accurate capsule polysaccharide synthesis and virulence in the yeast *Cryptococcus neoformans*. Eukaryotic cell *6*, 630-640.
Liu, T.B., Wang, Y., Baker, G.M., Fahmy, H., Jiang, L., and Xue, C. (2013). The glucose sensor-like protein Hxs1 is a high-affinity glucose transporter and required for virulence in *Cryptococcus neoformans*. PloS one *8*, e64239.
Mandel, M.A., Grace, G.G., Orsborn, K.I., Schafer, F., Murphy, J.W., Orbach, M.J., and Galgiani, J.N. (2000). The *Cryptococcus neoformans* gene *DHA1* encodes an antigen that elicits a delayed-type hypersensitivity reaction in immune mice. Infection and immunity *68*, 6196-6201.
Moyrand, F., Chang, Y.C., Himmelreich, U., Kwon-Chung, K.J., and Janbon, G. (2004). Cas3p belongs to a seven-member family of capsule structure designer proteins. Eukaryotic cell *3*, 1513-1524.
O'Meara, T.R., Hay, C., Price, M.S., Giles, S., and Alspaugh, J.A. (2010). Cryptococcus neoformans histone acetyltransferase Gcn5 regulates fungal adaptation to the host. Eukaryotic cell *9*, 1193-1202.
O'Meara, T.R., Holmer, S.M., Selvig, K., Dietrich, F., and Alspaugh, J.A. (2013). *Cryptococcus neoformans* Rim101 is associated with cell wall remodeling and evasion of the host immune responses. mBio *4*.
O'Meara, T.R., Xu, W., Selvig, K.M., O'Meara, M.J., Mitchell, A.P., and Alspaugh, J.A. (2014). The *Cryptococcus neoformans* Rim101 transcription factor directly regulates genes required for adaptation to the host. Molecular and cellular biology *34*, 673-684.
Wang, P., Perfect, J.R., and Heitman, J. (2000). The G-protein beta subunit *GPB1* is required for mating and haploid fruiting in *Cryptococcus neoformans*. Molecular and cellular biology *20*, 352-362.
